# Lynch Syndrome in Focus: A Multidisciplinary Review of Cancer Risk, Clinical Management, and Special Populations

**DOI:** 10.3390/cancers17243981

**Published:** 2025-12-13

**Authors:** Seyma Eroglu, Ilhan Birsenogul, Alexandra P. Bowen, Joseph F. Doyle, Stephen E. Pupkin, Joaquin Villar, Christopher M. Tarney, Edwin Gandia, Danielle M. Pastor, Juvianee I. Estrada-Veras

**Affiliations:** 1 Medical Oncology Service, Center for Cancer Research, National Cancer Institute, National Institutes of Health, Bethesda, MD 20892, USA; seyma.eroglu@nih.gov; 2 Independent Researcher, Bethesda, MD 20814, USA; ilhanbirsenogul@gmail.com; 3 The Henry M. Jackson Foundation for the Advancement of Military Medicine, Bethesda, MD 20817, USAstephen.e.pupkin.ctr@health.mil (S.E.P.); joaquin.villar.ctr@usuhs.edu (J.V.); juvianee.estrada-veras.ctr@usuhs.edu (J.I.E.-V.); 4 Department of Surgery, Uniformed Services University of the Health Sciences, Bethesda, MD 20814, USA; 5 Murtha Cancer Center, Walter Reed National Military Medical Center, Bethesda, MD 20814, USA; 6 The Defense Health Agency, Falls Church, VA 22042, USA; 7 Breast Care Center, Walter Reed National Military Medical Center, Bethesda, MD 20814, USA; 8 Center for Military Precision Health, Uniformed Services University of the Health Sciences, Bethesda, MD 20814, USA; 9 The American Genome Center, Bethesda, MD 20814, USA; 10 Medical Corps, US Army, Fort Sam Houston, TX 78234, USA; 11 Department of Gynecologic Surgery and Obstetrics, Uniformed Services University of the Health Sciences, Bethesda, MD 20814, USA; 12 Gynecologic Oncology, Walter Reed National Military Medical Center, Bethesda, MD 20814, USA; 13 Department of Pathology, Walter Reed National Military Medical Center, Bethesda, MD 20814, USA; 14 Sidney Kimmel Comprehensive Cancer Center, Johns Hopkins University School of Medicine, Baltimore, MD 21205, USA

**Keywords:** Lynch syndrome, mismatch repair genes, microsatellite instability, molecular diagnostics, Lynch-like syndrome, cancer genetics, cancer surveillance, risk-reduction strategies, immunotherapy

## Abstract

Lynch syndrome (LS), also known as hereditary nonpolyposis colorectal cancer (HNPCC), is a hereditary cancer syndrome significantly increasing the risk of colorectal cancer (CRC) and various extracolonic cancers, including endometrial, ovarian, and gastric cancers. LS results from germline pathogenic variants (GPVs) in DNA mismatch repair (MMR) genes, such as *MLH1*, *MSH2*, *MSH6*, and *PMS2*, leading to microsatellite instability (MSI). This review explores the multifaceted aspects of LS, covering clinical presentation, genetic underpinnings, and emerging therapeutic strategies. The discussion explores the importance of identifying at-risk individuals, facilitating personalized cancer surveillance and prevention strategies. Molecular insights into distinguishing between sporadic and LS-associated cancers are also examined, with a focus on somatic testing methods, including MSI and immunohistochemistry (IHC). The gynecological cancer risks, particularly those related to endometrial and ovarian malignancies, are addressed, underscoring the need for early detection and risk-reducing interventions. Recent advancements in the management of colorectal and other LS-related cancers are highlighted, with particular attention to the growing role of immunotherapy, including immune checkpoint inhibitors, and immunoprevention strategies. With ongoing advances in our understanding of LS, opportunities for earlier detection, more effective prevention, and innovative treatments continue to expand. This narrative review adopts a multidisciplinary approach to provide a comprehensive understanding of LS, from its genetic basis to current clinical and therapeutic practices, with the ultimate goal of improving patient outcomes and enhancing the quality of care.

## Simple Summary

Lynch syndrome (LS) is the most common autosomal dominant hereditary colorectal cancer syndrome, resulting from germline pathogenic variants in the DNA mismatch repair genes *MLH1*, *MSH2*, *MSH6*, *PMS2*, and deletions in *EPCAM*. The spectrum of tumors associated with LS is broad, and disease phenotype is quite variable, with risk of cancer development largely dependent on the involved gene. Accurate diagnosis through genetic testing and comprehensive family history assessments to identify at-risk individuals is critical to the facilitation of personalized cancer surveillance and prevention strategies. This narrative review aims to provide a comprehensive understanding of LS, from its molecular basis to current guidelines informing clinical and therapeutic practices.

## 1. Overview of Lynch Syndrome

### 1.1. Historical Background

The publication of the pedigree of “Family G” by Dr. Aldred Scott Warthin [1], a pathologist working at the University of Michigan in 1913, provided insights into hereditary factors in cancer, particularly in relation to colorectal cancer (CRC). This family’s pedigree was characterized by a distinct susceptibility to the development of endometrial cancer (EC) and gastric cancer (GC), showing an autosomal dominant (AD) pattern of inheritance. His work introduced the notion of hereditary predisposition to cancer. Dr. Warthin’s findings were further described by Dr. Henry T. Lynch, who described two families (N-Nebraska and M-Michigan) in 1966 [2]. The families were affected by colon, gastric, and endometrial cancers. Dr. Lynch then proposed that an autosomal dominant “cancer family syndrome” was responsible for the cancers in the described families [3]. Years later, in 1984, the family syndrome was called Lynch syndrome (LS) in honor of Dr. Lynch’s efforts throughout the years in expanding the understanding of the role of heredity in cancer, and how this can impact cancer prevention and management [4].

### 1.2. Etiology and Prevalence

LS is one of the most common autosomal dominant cancer predisposition syndromes, affecting approximately 1 in 279 individuals (0.36%) [5]. It is the leading hereditary cause of CRC and EC, accounting for about 3% of cases in each [6,7]. LS results from heterozygous germline pathogenic variants (GPVs) in DNA mismatch repair (MMR) genes (*MLH1*, *MSH2*, *MSH6*, *PMS2*), 3′ deletions in the *EPCAM* gene, or a constitutional *MLH1* epimutation, which is characterized by methylation of one promoter allele resulting in transcriptional silencing of that affected allele in normal somatic tissues, while the gene itself remains structurally intact [8]. Among these, *PMS2* and *MSH6* variants are the most prevalent (1 in 714 and 1 in 758, respectively), while *MLH1* and *MSH2* variants are less common (1 in 1946 and 1 in 2841) [5].

### 1.3. Molecular Pathogenesis of LS

MMR is a highly conserved biological pathway that plays a key role in maintaining genomic stability by correcting base–base mismatches and insertion/deletion mispairs generated during DNA replication and recombination. Several human MMR proteins have been identified based on their homology to *E. coli* MMR proteins (Figure S1) [9]. These include human homologs of MutS, MutL, EXO1, single-strand DNA-binding protein RPA, proliferating cell nuclear antigen (PCNA), DNA polymerase δ (pol δ), and DNA ligase I [10].

The protein hMSH2 heterodimerizes with hMSH6 or hMSH3 to form hMutSα or hMutSβ, respectively, both of which are ATPases that play a critical role in mismatch recognition and initiation of repair [11]. hMLH1 heterodimerizes with hPMS2, hPMS1, or hMLH3 to form hMutLα, hMutLβ, or hMutLγ, respectively. hMutLα is the only one required for MMR [11]. hMutLα possesses an ATPase activity, and defects in this activity inactivate MMR in human cells. To date, only pathogenic variants (PVs) in the MMR genes (*MLH1*, *MSH2*, *MSH6*, or *PMS2*) or in the *EPCAM* gene have been associated with LS [12].

Deficiency in DNA MMR (dMMR) leads to microsatellite instability (MSI), characterized by variably sized repetitive DNA sequences, or microsatellites, fostering a hypermutable, tumor-promoting environment [13]. Although microsatellites are widespread in the human genome, LS-associated dMMR mainly causes CRC and EC. The predominance of these cancers in LS is multifactorial, and further studies are warranted.

- High epithelial turnover: These tissues exhibit rapid epithelial cell turnover, increasing the frequency of replication errors. In the absence of functional MMR, such errors accumulate, leading to increased genomic instability and cancer development [14].
- Tumor type-specific target gene mutations: dMMR generates many MSI-driven frameshift mutations that can inactivate tumor-suppressive functions. The most frequently affected genes are different by tumor type, conferring selective advantages in CRC (e.g., *TGFBR2*, *ACVR2A*, *BAX*) versus EC (e.g., *JAK1*, *TFAM*) [15].
- Dietary mutagens: Certain dietary mutagens—especially 2-amino-1-methyl-6-phenylimidazo [4,5-b]pyridine (PhIP), a heterocyclic aromatic amine formed in meat cooked at high temperatures—have been shown to promote CRC development in LS through several mechanisms. These include facilitating the loss of the remaining wild-type MMR allele, impairing protective DNA damage signaling in the resulting dMMR cells, and exacerbating mutability, ultimately generating a compound hypermutator phenotype [16].

## 2. Molecular Genetics of Lynch Syndrome and Associated Genes

### 2.1. MLH1 (MIM#120436)

Located in chromosome 3 (3p22.2), *MLH1* heterodimerizes with *PMS2* to form MutL alpha. It spans 19 exons. 15–40% of LS are attributed to PVs in *MLH1*. The majority of the PVs in this gene are truncating variants (frameshift, nonsense, splicing, large deletions or duplications), but missense variants affecting its interaction with *PMS2* are also frequent. Balanced inversions affecting *MLH1* have been described [17]. In addition, constitutional epimutation of *MLH1* has been identified as an alternative mechanism that predisposes to LS [18]. Founder mutations have been described in the literature, including an exon 16 deletion caused by an Alu-mediated recombination event in the Finnish population and a frameshift variant segregating with a haplotype in northern Italy [19,20]. At older ages, *MLH1* mutation carriers exhibit increased risks of urinary tract and upper gastrointestinal cancers, including gastric and small bowel cancers [21]. Truncating and missense or aberrant splicing PVs were associated with similar average cumulative incidences of cancer in carriers of *MLH1* PVs [22].

### 2.2. PMS2 (MIM#600259)

Located in chromosome 7 (7p22.1), *PMS2* spans 15 exons. 5–25% of LS are attributed to PVs in *PMS2*. Clinical testing of *PMS2* is notoriously difficult to analyze due to the existence of multiple pseudogenes [23]. Most PVs in this gene are truncating, including frameshift, nonsense, splicing, gross deletion/duplication. Missense PVs have also been reported. Recurrent and founder mutations appear to be common in *PMS2* [24].

### 2.3. MSH6 (MIM#600678)

Located in chromosome 2 (2p16.3), *MSH6* spans 10 exons. It heterodimerizes with *MSH2* to form MutS alpha. 12–35% of LS are attributed to PVs in *MSH6*. The spectrum of PVs in *MSH6* includes null alleles, DNA sequence variations that result in no functional gene product, as well as missense variants affecting DNA MMR. Founder mutations in the Ashkenazi Jewish population have been described [25]. Female carriers of a PV in *MSH6* are at high risk of EC compared with other organs. CRC risk is lower than that in carriers of a PV in *MLH1* or *MSH2*. Because a PV in *MSH6* causes a sex-limited trait with high penetrance in females compared with males, this limits the utility of family history for identifying *MSH6*-associated LS [21].

### 2.4. MSH2 (MIM#609309)

Located in chromosome 2 (2p21-p16.3), *MSH2* spans 16 exons. 20–40% of LS are attributed to PVs in *MSH2*. The spectrum of PVs observed in *MSH2* is very similar to the one found in *MLH1*, and includes missense variants, truncating variants, exonic deletion/duplication, and structural variants. A recurrent inversion affecting exons 1–7 has been described [26]. There are multiple founder mutations described in the literature. The c.942+3A>T variant is very challenging to detect by sequencing because it lies adjacent to a poly(A) tract, which is a stretch of consecutive adenine nucleotides and can cause sequencing errors. Similar to *MLH1*, truncating and missense or aberrant splicing PVs were associated with similar average cumulative incidences of cancer in carriers of *MSH2* PVs [22].

### 2.5. EPCAM (MIM#185535)

This gene is located only 15 kb upstream of *MSH2*. Deletions encompassing the 3′ end of *EPCAM* without affecting the open reading frame of *MSH2* result in epigenetic silencing of *MSH2* via transcriptional read-through, resulting in promoter hypermethylation and allele-specific gene silencing of *MSH2* in *EPCAM*-expressing tissues [27]. Deletions of *EPCAM* are associated with early-onset CRC but rarely with extra-gastrointestinal tumors.

### 2.6. Variant Interpretation

The majority of PVs in MMR genes are loss-of-function (LOF) variants, which include frameshift, nonsense, canonical splice variants, and copy number variants. In addition, balanced inversions have been described in both *MLH1* and *PMS2* [17,26], which are challenging to detect because they do not alter DNA copy number or sequence, making them undetectable by standard sequencing and copy number assays, and requiring specialized structural variant analysis for identification. Variant interpretation for these LOF variants following the American College of Medical Genetics (ACMG) and Association for Molecular Pathology (AMP) guidelines is relatively straightforward since LOF variants in these four genes (*MLH1*, *MSH2*, *MSH6*, and *PMS2*) are known to be pathogenic [28]. There are, however, thousands of missense variants classified as a variant of uncertain significance (VUS) in databases such as ClinVar and InSiGHT [28,29]. Functional studies to determine whether VUSs are pathogenic or benign have been described [30,31,32,33], but additional work regarding the area of reclassification is needed.

It is generally recommended that VUSs detected in hereditary cancer settings be managed as if results were established to be negative *unless proven otherwise*, as these VUSs are very frequently reclassified as non-pathogenic in the future. The potential for negative effects in misinterpretation with VUSs should not be understated: patients can experience significant morbidity, including unnecessary prophylactic surgeries, such as hysterectomy, when VUSs are misinterpreted as positive results. It is critical that clinicians ordering genetic testing familiarize themselves with VUS result interpretation and engage with genetics clinical services when ordering testing in order to avoid such outcomes.

## 3. Nomenclature: Lynch Syndrome and Its Mimickers

The terminology surrounding LS has evolved alongside advances in genetic understanding. Historically, the term hereditary nonpolyposis colorectal cancer (HNPCC) was used interchangeably with LS and was based primarily on clinical criteria like the Amsterdam I/II and Bethesda guidelines [34,35]. However, following the 2004 International Bethesda Meeting, “Lynch syndrome” became the preferred term to reflect the broader tumor spectrum associated with the condition [36].

Today, LS is defined at the molecular level by heterozygous GPVs in MMR genes. While the term “HNPCC” is still used in some contexts, it now refers more broadly to nonpolyposis hereditary colorectal cancers, including LS [37]. For consistency, we retain “HNPCC” when referencing historical criteria, although “LS” is now the standard term.

### 3.1. Familial Colorectal Cancer Type X (FCCTX)

Familial colorectal cancer type X describes families that meet Amsterdam I criteria but lack dMMR and identifiable GPVs in MMR genes [38]. Unlike LS, FCCTX has no established molecular basis and likely represents a heterogeneous clinical entity. Proposed associated genes include *BMPR1A* [39], *RPS20* [40], *SEMA4A* [41], and *SETD6* [42], among others [43,44,45,46,47], though none are definitive. Compared with LS, it was shown that FCCTX exhibits a predilection for rectal cancer and a higher prevalence among male patients, along with moderate penetrance characterized by later onset and fewer synchronous or metachronous CRCs. It also displays distinct pathological features, including lower tumor grade and fewer mucinous adenocarcinomas [48]. The term FCCTX currently serves as a clinical placeholder for cases without a known genetic cause, but ongoing genomic advances may allow more precise molecular classification in the future.

### 3.2. Lynch-like Syndrome (LLS)

Lynch-like syndrome (LLS) describes cases with dMMR tumors but no detectable GPVs in MMR genes. These cases pose a clinical challenge, as there is no consensus on their management, and understanding the underlying mechanisms is essential to guide care. Potential etiologies of LLS include the following:

- Biallelic somatic mutations: Identified in 69% of patients with dMMR tumors and negative germline MMR testing [49], and similarly reported in 88.4% of patients with dMMR tumors and negative multigene panel testing (MGPT) [50].
- Undetected germline variants in MMR genes: Some variants may remain undetected due to limitations in current sequencing technologies.
- GPVs in non-MMR genes: Found in 4.5% of patients with dMMR tumors not explained by *MLH1* hypermethylation [50].
- Mosaic LS: Rare cases of somatic mosaicism, presence of two or more genetically distinct cell populations, in MMR genes have been reported, often involving multiple LS-associated cancers [51,52,53,54,55]. Walker et al. confirmed a low-level mosaic *MSH6* variant using droplet digital polymerase chain reaction (ddPCR) across colonic mucosa, saliva, and blood after identifying a shared somatic *MSH6* mutation in both colorectal and endometrial tumors [56]. O’Brien et al. described a mosaic *MSH2* PV in a patient with endometrial adenocarcinoma and keratoacanthomas (KA); the diagnosis was confirmed through deep tumor sequencing and reanalysis of blood-derived DNA, following IHC that revealed loss of *MSH2* and *MSH6* expression in both tumors [55].

Given the diverse etiologies and their implications for management, surveillance, and counseling, the continued use of umbrella terms like “Lynch-like syndrome” or “mutation-negative Lynch syndrome” may lead to clinical confusion [57]. In select cases, somatic tumor sequencing and broader germline MGPT can help clarify the diagnosis [49,50], though these tools should be applied judiciously. Broader testing increases the likelihood of identifying VUSs, which can complicate interpretation, raise patient anxiety, and increase cost. The tailoring of testing strategies to the clinical context can help support more personalized care [58]. The NCCN Clinical Practice Guidelines in Oncology (NCCN Guidelines^®^) recommend providing care based on personal and family history until further research on Lynch-like syndrome emerges [59].

Variants of LS, such as Muir-Torre and Turcot syndromes, were historically defined based on distinctive clinical features: sebaceous neoplasms (SNs) in Muir-Torre, and glioblastomas in Turcot syndrome [60,61]. These are now recognized as phenotypic manifestations within the LS spectrum rather than distinct syndromes [62,63]. The term “Turcot syndrome” is also used to describe a variant of familial adenomatous polyposis (FAP) with associated brain tumors, prompting the NCCN Guidelines^®^ to recommend against its use due to ambiguity [59].

### 3.3. Constitutional Mismatch Repair Deficiency

Constitutional mismatch repair deficiency (CMMRD) is a rare, childhood-onset cancer predisposition syndrome resulting from biallelic GPVs in MMR genes and inherited in an autosomal recessive pattern. PVs in *PMS2* are the most common cause of CMMRD, likely due to the lower penetrance of heterozygous *PMS2* mutation carriers compared to other Lynch-associated genes [64]. Individuals with CMMRD lack a functional MMR system in all cells, in contrast to LS, where dMMR results from somatic inactivation of the second allele. The cancer spectrum includes hematologic malignancies, high-grade gliomas, and early-onset CRCs, often with multiple gastrointestinal adenomas during adolescence [65]. Non-neoplastic features include café-au-lait macules mimicking Neurofibromatosis type 1 and brain developmental venous anomalies [65]. The incidence is ~1 in 1 million but is higher in populations with consanguinity or founder variants [65]. ERN GENTURIS guidelines recommend testing partners of LS carriers or CMMRD patients if any of the following apply: consanguinity, founder population background, or a family history suggestive of LS [65], and such testing should be preceded by ethical considerations and pretest counseling.

### 3.4. Double Heterozygosity in LS

Double heterozygosity in LS, where an individual carries PVs in two different MMR genes, is rare and has not been shown to result in CMMRD [66]. In a cohort of over 75,000 cases tested for two or more MMR genes at a clinical diagnostic laboratory, only seven LS double heterozygotes were identified. None of these patients’ histories showed classic features of CMMRD [66]. Further studies are warranted to determine the relative cancer risk among LS double heterozygotes.

## 4. Identifying Individuals at High Risk for Lynch Syndrome: Navigating the Lack of Consensus Criteria

### 4.1. Clinical Criteria

**Figure 1:**
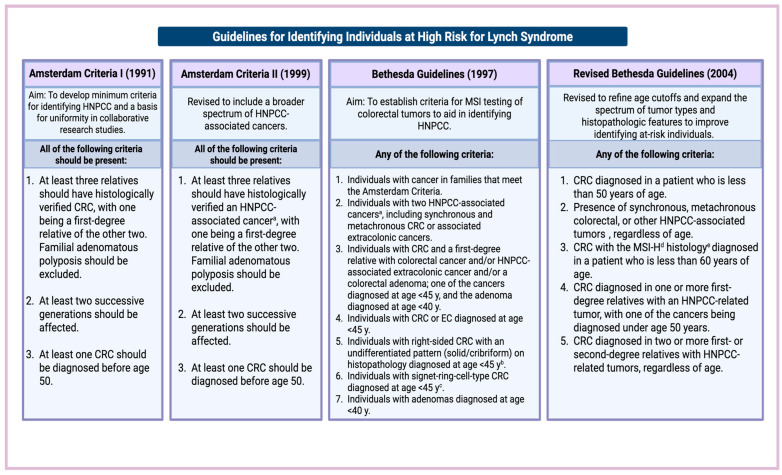
Clinical guidelines for identifying individuals at high risk for Lynch Syndrome [34,35,36]. Abbreviations: HNPCC, hereditary nonpolyposis colorectal cancer; CRC, colorectal cancer; EC, endometrial cancer; MSI-H, microsatellite instability-high. ^a^ The term “HNPCC” is used to reflect the terminology in the original clinical criteria. Over time, “HNPCC” has been replaced by “LS”, reflecting an evolved understanding of the disease. ^b^ Solid/cribriform, defined as poorly differentiated or undifferentiated carcinoma composed of irregular, solid sheets of large eosinophilic cells and containing small gland-like spaces. ^c^ Composed of >50% signet ring cells. ^d^ MSI-H refers to changes in two or more of the five National Cancer Institute-recommended panels of microsatellite markers. ^e^ Presence of tumor-infiltrating lymphocytes, Crohn’s-like lymphocytic reaction, mucinous/signet-ring differentiation, or medullary growth pattern. Created in BioRender. Eroglu S. (2025). https://BioRender.com/4x17llr (accessed on 2 November 2025).

In 1991, the International Collaborative Group on HNPCC (ICG-HNPCC) introduced the Amsterdam criteria to standardize LS patient selection in research, prioritizing specificity (Figure 1) [34]. However, these criteria excluded many true LS cases due to their narrow focus on CRC and larger kindreds. In response, the National Cancer Institute convened a 1996 workshop in Bethesda, leading to the development of the Bethesda guidelines, which broadened identification by recommending MSI testing for certain tumors [67]. In 1999, the Amsterdam criteria were revised (Amsterdam II) to include extracolonic LS-associated cancers [35]. The Amsterdam I criteria demonstrated a sensitivity of 61% and a specificity of 67%, while Amsterdam II improved sensitivity to 78% with a similar specificity (61%). Among the criteria evaluated, the Bethesda guidelines were the most sensitive (94%) but had the lowest specificity (25%) [68].

In 2004, the Bethesda guidelines were revised to further expand the clinical identification of individuals at risk for LS [69]. In a study of 500 CRC patients, 18 of whom were diagnosed with LS via tumor testing, only 39% met Amsterdam II criteria, 72% met the revised Bethesda guidelines, and 28% met neither. These findings underscore the limitations of relying solely on family history to identify LS cases [70].

### 4.2. Tumor Testing

**Figure 2:**
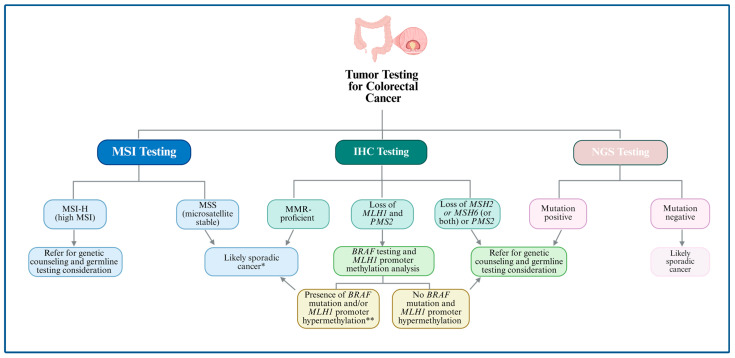
Tumor testing algorithm for Lynch Syndrome in patients with colorectal cancer. Note: Tumor testing for MMR deficiency can be initiated using either immunohistochemistry (IHC), microsatellite instability (MSI) testing, or next-generation sequencing (NGS), depending on local practice and assay availability. * Caution is warranted, as 10% of CRC patients with LS have been shown to be MMR-proficient (pMMR) [73]. ** Results should be interpreted carefully, especially in younger patients or those with a strong family history of cancer. Created in BioRender. Eroglu, S. (2026) https://BioRender.com/gn11wfu (accessed on 6 November 2025).

Due to the suboptimal sensitivity of most clinically based criteria for identifying individuals with LS, several groups have evaluated an alternative strategy known as universal screening, in which all individuals newly diagnosed with CRC undergo IHC staining for loss of one of the four DNA MMR proteins, and/or MSI by polymerase chain reaction (PCR) or a validated MSI assessment by next-generation sequencing (NGS) assays (Figure 2) [71]. This approach has demonstrated a sensitivity of 100% and a specificity of 93.0% for detecting LS [72]. An alternative strategy involved testing all CRC patients diagnosed before age 70, as well as older patients who meet the revised Bethesda guidelines [72]. This selective approach yielded a sensitivity of 95.1% and a specificity of 95.5%, representing higher specificity than universal screening while maintaining better sensitivity than the Bethesda criteria alone [72].

The cost-effectiveness of universal tumor screening (UTS) has been established and endorsed by multiple organizations, including the 2009 Evaluation of Genomic Applications in Practice and Prevention (EGAPP) Working Group at the Centers for Disease Control and Prevention, the 2013 European Society for Medical Oncology (ESMO), and the 2014 United States (U.S.) Multi-Society Task Force on Colorectal Cancer [74,75,76,77]. In 2016, the NCCN Guidelines recommended universal screening for all patients with CRCs and ECs to increase sensitivity for LS detection [78,79].

In addition to serving as critical biomarkers in LS screening, tumor dMMR and MSI status also carry significant therapeutic implications. In 2017, the U.S. Food and Drug Administration (FDA) granted tissue site-agnostic approval for pembrolizumab for the treatment of all advanced microsatellite instability-high (MSI-H)/dMMR solid tumors [80], leading to increased MMR/MSI testing across various cancer types. According to College of American Pathologists guidelines, it is strongly recommended that pathologists use MMR-IHC and/or MSI by PCR to detect DNA MMR defects in patients with CRC who are being considered for immune checkpoint inhibitor (ICI) therapy [71]. In addition to UTS for all patients with CRCs and ECs by MSI or IHC testing to detect loss of one of the four DNA MMR proteins, the NCCN Guidelines recommend considering tumor screening for dMMR in SN and the following adenocarcinomas: small bowel, ovarian, gastric, pancreatic, biliary tract, brain, bladder/urothelial, and adrenocortical cancers, regardless of age at diagnosis [59].

A comprehensive study analyzing more than 15,000 tumors across 50 cancer types revealed that 16% of patients with MSI-H tumors have LS [81]. Notably, 45% of LS patients with MSI-H or intermediate MSI (MSI-I) tumors did not meet clinical criteria for genetic testing based on personal or family cancer history [81]. This finding underscores the limitations of relying solely on clinical criteria for genetic testing and highlights the importance of UTS to ensure that at-risk individuals are identified, particularly those who might otherwise be missed under traditional guidelines.

However, such testing often does not lead to germline testing for LS. Despite guidelines strongly recommending LS screening for MSI-H tumors due to its critical implications for cancer surveillance, risk-reducing surgeries, and familial risk assessment, Papadopoulou et al. (2024) reported that up to 77% of patients with MSI-H tumors do not undergo the recommended genetic testing for LS [82]. Several barriers have been reported as contributing to the underutilization of LS genetic testing. Many patients prioritize identifying the most effective cancer treatment and often decline testing due to anxiety about receiving a positive result and concerns about the cost of analysis [82]. However, lowering the price of testing has had little impact on uptake [82], suggesting that financial constraints alone do not fully explain the low testing rates. Another potential factor is the lack of public awareness about the benefits of hereditary cancer analysis. These findings highlight the need for improved patient education and better integration of genetic counseling into oncology care to ensure that at-risk individuals receive appropriate genetic evaluation.

Furthermore, UTS has its limitations. In a study by Pearlman et al., UTS alone was found to be insufficient for identifying all CRC patients with hereditary syndromes, including LS, as 6.3% (9 of 144) of LS cases would have been missed if UTS had been the sole method for germline assessment [50]. The study demonstrated that the missed LS cases included six patients with MMR-proficient (pMMR) tumors, two with constitutional *MLH1* hypermethylation, and one with *MLH1*/*PMS2* loss accompanied by *MLH1* hypermethylation [50].

**Figure 3:**
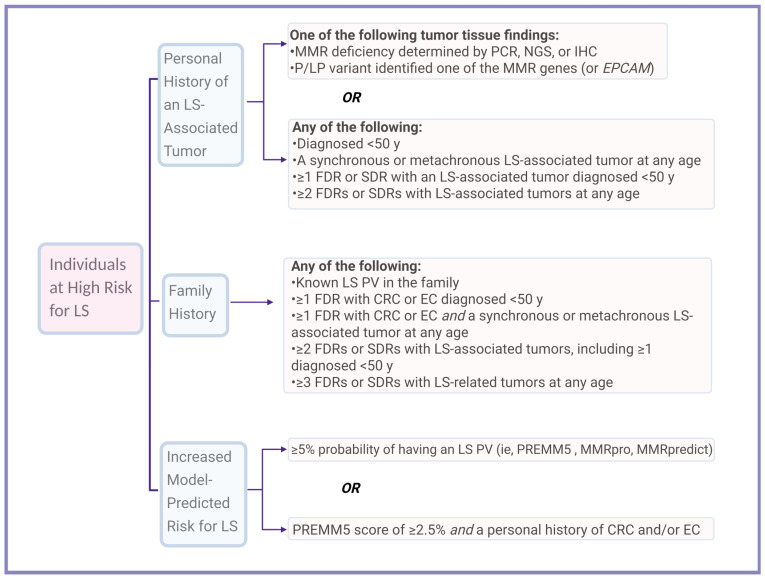
Algorithm for identifying individuals at high risk for Lynch Syndrome. Abbreviations: LS, Lynch syndrome; MMR, mismatch repair; P/LP, pathogenic/likely pathogenic; PCR, polymerase chain reaction; NGS, next-generation sequencing; IHC, immunohistochemistry; PV, pathogenic variant; FDR, first-degree relative; SDR, second-degree relative; CRC, colorectal cancer; EC, endometrial cancer. Adapted with permission from the NCCN Clinical Practice Guidelines in Oncology (NCCN Guidelines^®^) for Genetic/Familial High-Risk Assessment: Colorectal, Endometrial, and Gastric V.1.2025. © 2025 National Comprehensive Cancer Network, Inc. All rights reserved. The NCCN Guidelines^®^ and illustrations herein may not be reproduced in any form for any purpose without the express written permission of NCCN. To view the most recent and complete version of the guideline, go online to NCCN.org. The NCCN Guidelines are a work in progress that may be refined as often as new significant data becomes available. NCCN makes no warranties of any kind whatsoever regarding their content, use, or application, and disclaims any responsibility for their application or use in any way. Created in BioRender. Eroglu S. (2025). https://BioRender.com/n23t724 (accessed on 2 November 2025).

Due to the limitations of individual testing methods, several national and international guidelines now recommend a more inclusive approach to LS testing, incorporating personal and family history of cancer, tumor-based screening, and model-predicted risk estimates (Figure 3) [59].

### 4.3. Prediction Models

Several prediction models, including PREMM5, MMRpro, and MMRpredict, have been developed to estimate the likelihood of carrying a GPV in MMR genes, based on personal and family cancer history (Table 1) [83,84,85]. These tools support clinical decision-making and may help prioritize patients for genetic evaluation, particularly in resource-limited settings where tumor testing may not be readily available [86]. While useful, their accuracy relies on detailed personal and family history of cancer, which may be incomplete, and their cost-effectiveness compared to tumor testing remains unclear.

The NCCN Guidelines recommend genetic testing for individuals with a ≥5% risk of having an MMR gene PV based on predictive models (e.g., PREMM5, MMRpro, MMR-predict) [59]. Additionally, individuals with a personal history of CRC and/or EC who have a PREMM5 score of ≥2.5% should be considered for MGPT (Table 1, Figure 3) [59].

### 4.4. Germline Testing

Germline testing typically starts with an individual who has been diagnosed with an LS-associated tumor. Nonetheless, in some families, when an affected relative is not living or willing to undergo testing, an unaffected family member may be tested first. Single-gene testing or MGPT can be considered as initial testing strategies. MGPT that includes *MLH1*, *MSH2*, *MSH6*, and *PMS2*, as well as *EPCAM* deletion analysis, is recommended over targeted gene testing because of overlapping clinical features, higher PV detection rates, and improved precision in clinical decision-making [87]. MGPT not only limits the identification of VUS in genes that do not explain the phenotype [87] but also enables the detection of the most complex variants described in these genes.

Constitutional *MLH1* promoter hypermethylation is rare and cannot be detected by either sequencing or copy number analysis of the *MLH1* gene. DNA methylation studies of the *MLH1* promoter are required for the diagnosis. Most cases arise de novo; therefore, a significant family history of cancer may be absent. The NCCN Guidelines recommend consideration of referral to a clinician with expertise in genetic testing for evaluation of constitutional *MLH1* methylation testing in patients with early-onset CRC (≤55 years), no *BRAF* V600E PV, loss of *MLH1* on IHC, and no *MLH1* GPVs, or more than one tumor with *MLH1* promoter hypermethylation at any age [59,88].

Although exome and genome sequencing are not the recommended strategies for individuals undergoing evaluation for possible LS, MMR genes are included in the ACMG SF v3.3 list for reporting secondary findings in clinical exomes and genomes [89]. Given the high prevalence of LS (1/279) [5], the possibility of finding PVs/LPVs in these genes is relevant [90], even in families with no known LS diagnosis.

## 5. Tumor Spectrum in Lynch Syndrome

LS is associated with a broad spectrum of tumors (Table 2), including colorectal, endometrial, gastric, ovarian, pancreatic, urothelial, brain (typically glioblastomas), biliary tract, small intestine, and certain skin cancers (e.g., sebaceous adenomas (SA), sebaceous carcinomas (SC), and KA). However, the inclusion of some extracolonic cancers within the LS spectrum remains debated. For instance, while GeneReviews and European guidelines from the European Hereditary Tumour Group and the European Society of Coloproctology include prostate cancer, the NCCN Guidelines do not [37,91]. This discrepancy underscores the limitations of rigid, guideline-based criteria for genetic testing, as overly strict inclusion standards may exclude at-risk families from appropriate evaluation. Tumor-specific surveillance, chemoprevention, and risk-reduction strategies in LS are outlined in Table 3, comparing recommendations across five established clinical guidelines.

### 5.1. Colorectal Cancer

#### 5.1.1. Epidemiology for LS-Associated Colorectal Cancer

LS is present in about 1 in every 35 patients with colon cancer [70]. Neoplasms in this setting are associated with early-onset CRC, with individuals typically developing malignancy before the age of 50 years, although the estimated average age of presentation depends on the gene involved. Disease phenotype is quite variable, with the extent of risk of developing CRC also dependent on the particular gene involved (Table 2).

#### 5.1.2. Pathogenesis of LS-Associated Colorectal Cancer

LS-associated CRC is often proximal in location, although 40% of CRCs in this setting are found in the left colon or rectum [95,96,97]. Synchronous and metachronous tumors are also characteristic of the syndrome [98]. A pathophysiologic hallmark of CRC development in this setting is the acceleration of malignant degeneration of the precursor lesion, hence the importance of appropriate counseling and regular colonoscopy surveillance in this population [99].

Precursor lesions are polyps, which tend to be adenomatous but frequently display histologic features of mucinous villous growth patterns and moderate to high dysplasia. Tumors are often characterized by signet ring cells and a “Crohn’s-like” reaction, with an increased density of tumor-infiltrating lymphocytes and peritumoral lymphoid nodules. These malignancies tend to be poorly differentiated [100,101].

Although the polyp has long been widely regarded as the principal precursor lesion in LS-CRC carcinogenesis, alternative theories have been proposed in recent years to account for a subset of tumors that develop in the setting of LS, despite individual compliance with proper interval endoscopic surveillance. Ahadova et al. propose that this subset may develop “de novo” from MMR-deficient crypt foci, which are characterized by accelerated invasive growth from non-polypoid mucosa and may be influenced by the presence of mutations of the *CTNNB1* gene [102,103]. The *CTNNB1* gene is not only involved in the Wnt signaling pathway, but has also been demonstrated to be involved in hereditary MSI cancer development [104,105]. This proposed theory may account for the fraction of LS-CRCs that develop submucosally and may evade detection via colonoscopic visualization. Briefly, Adhova and co-investigators provide three potential mechanisms of carcinogenesis for LS-CRC: progression from pMMR adenomas; progression from dMMR crypt foci with development of adenoma followed by accelerated growth; and progression from dMMR crypt foci with development of invasive cancer in the absence of polyp formation [106]. While further evaluation is needed, this theory may provide plausible support for the underlying pathogenesis of LS-CRCs that arise despite vigilant adherence to surveillance recommendations and guidelines.

#### 5.1.3. Surveillance of LS-Associated Colorectal Cancer

Colonoscopic surveillance is critical for risk reduction of CRC development in this predisposed population. Guidelines for risk management are gene-specific, with the frequency of interval colonoscopy dictated by the affected gene involved. Aspirin chemoprevention may also be considered as a prevention strategy, as well as prophylactic surgery in certain cases; however, the type and extent of surgical resection should be specifically tailored to the individual and should take into consideration polyp burden and pathology, gene involvement, patient operative candidacy, and overall risk factors, as well as personal preferences. Individuals should understand that optimal surveillance modalities may change, and bowel function may be altered subsequent to the procedure performed. Surveillance recommendations as issued by various organizations and societies are delineated in Table 3.

#### 5.1.4. Immunotherapy in LS-Associated Colorectal Cancer

The vast majority of LS-CRCs exhibit dMMR and varying levels of MSI, resulting from the high mutational burden in genes encompassing coding microsatellites. The tumor microenvironment (TME) comprises pro-inflammatory cytokines and a dense infiltration of T cells lymphocytes, capable of executing heightened T cell responses [107,108]. A complex relationship exists between LS-CRC and the constituents of the associated TME, and contributes to the maintenance of balance between tumor elimination and development. Interestingly, over half of LS adenomas possess *TGFBR2* gene mutations [109]. The presence of these mutations in such precursor lesions supports the concept that immunoediting plays a role in early phases in the progression of adenoma to carcinoma, as well as in later stages [109].

Tumor mutational burden and high levels of MSI are thought to be predictive factors regarding enhanced cancer susceptibility to immunotherapeutic approaches. These features, as well as the increased neoantigen load, high density of infiltrating immune cells in the tumor microenvironment, and numerous immune-related genes found to be upregulated in LS-CRC have not only made this type of cancer a “prototype” through which to study the mechanisms of immunotherapy, but may also enable greater understanding regarding the sensitization to this modality of therapy by more “immunedormant” pMMR/microsatellite stable (MSS) CRC that comprise the majority of all CRC. Moreover, these features may afford an opportunity to employ immune-mediating agents in a prophylactic approach in those with LS.

### 5.2. Small Bowel Cancer

Small bowel adenocarcinoma (SBA) is a relatively rare but clinically significant manifestation of LS. dMMR is found in 21–26% of SBA cases, and LS accounts for 6–10% of all SBA patients [110,111,112,113]. *MSH2* and *MLH1* mutations are most common; *PMS2* mutations are less frequent [110,111,113,114]. LS-associated SBA tends to occur at a younger age (median 47.5–57 years) and more often affects males [110,111,112,113,114]. Tumors predominantly involve the duodenum (45–61%) and jejunum (15–56%), with rare ileal cases. Most are moderately differentiated and diagnosed at stage II or III [110,112,113].

One study identified six patients with SBA who met clinical criteria for LS prior to diagnosis, but none had undergone genetic testing. An additional three patients had suggestive family histories but did not meet formal criteria [110]. These observations not only highlight the limitations of relying solely on clinical criteria, since even eligible patients may remain undertested, but also underscore the importance of universal MMR screening to improve diagnosis, guide treatment decisions, and identify LS families for cascade testing and surveillance [110,111].

### 5.3. Gastric Cancer

Gastric cancer (GC) is an infrequent but recognized malignancy in LS, with a reported prevalence of 0.9–3.7% [114,115,116,117,118]. *MSH2* and *MLH1* mutations are most commonly involved, while *MSH6* and *PMS2* mutations occur less frequently [114,115,116,117]. Most studies report a male predominance. The median age at diagnosis ranges from 51 years to 56.5 years in cohorts with surveillance, compared to 68 years when screening is not routinely performed.

A first-degree family history is present in 18–32% of LS-associated GC cases [114,115,117]. Risk factors include male sex, older age, and family history [115]. *Helicobacter pylori* infection is reported in 17–75% of cases. Histologic subtypes are predominantly intestinal (57%) and diffuse (29%), with occasional mixed or mucinous types [118].

Surveillance studies support the utility of esophagogastroduodenoscopy (EGD). In one cohort of 323 asymptomatic LS patients, clinically actionable findings were identified in nearly 18%, including five upper gastrointestinal cancers [119]. Kim et al. recommend EGD every 3 years, or 1–2 years in those with additional risk factors [115]. Kumar et al. found that 80% of surveillance-detected GCs were stage I, despite negative EGDs within the prior two years, supporting an interval of 1–2 years [116]. Ladigan-Badura et al. identified GC before age 40 in 10.6% of patients, supporting EGD initiation by age 30, in conjunction with colonoscopy every 1–2 years [117]. In contrast, a retrospective cohort study evaluating the clinical data of individuals with LS who had not undergone routine surveillance via EGD, but were registered in the Dutch Hereditary Cancer Registry, demonstrated a very low cumulative incidence (≤1%) of GC in individuals under the age of 50 years [118]. Moreover, of all GCs diagnosed in the total population, 32% were stage I at diagnosis, and 62% were resectable; these findings highlight the importance of individualized assessment and heightened clinical awareness, suggesting that symptom-driven endoscopy may be an alternative to regularly scheduled interval EGD surveillance for this population.

### 5.4. Gynecologic Cancers

Women with LS are at elevated risk for both EC and ovarian cancer (OC). EC is the most common extracolonic cancer associated with LS and is often the initial presenting malignancy in women [120]. LS is also the second most common cause of inherited OC, following hereditary breast and ovarian cancer (HBOC) syndrome [121]. Women with LS who develop EC or OC are diagnosed at younger ages than the average population, with a mean age of 47–49 years for EC and 42–49 years for OC [122,123,124]. Regarding prognosis, it is controversial whether LS-associated EC has a worse prognosis, whereas for OC, women with LS tend to present with earlier stage cancer with an overrepresentation of endometrioid and clear cell histologies compared to the general population [124,125].

The risk for cancer depends on the MMR mutation (Table 2). Notably, the risk for OC in women with PV in *MSH6* and *PMS2* is not much higher than the general population, which is important when considering screening and preventative options (Table 3).

#### 5.4.1. Surveillance for LS-Associated Gynecologic Malignancies

- Endometrial cancer

Women with LS should be educated on warning symptoms for EC, such as abnormal uterine bleeding or postmenopausal bleeding, and counseled that these symptoms require evaluation [59,125]. Women with LS presenting with either a change in their normal bleeding pattern or with postmenopausal bleeding should undergo endometrial sampling with an endometrial biopsy [59,125]. Although some studies have shown that EC screening may not reduce morbidity or mortality in women with LS [126], endometrial biopsies can be considered, given the early age at which these malignancies often occur. Endometrial biopsy is a highly sensitive and specific test and, thus, screening every 1–2 years starting at age 30–35 years can be considered (Table 3) [59,125]. The use of routine transvaginal ultrasound in postmenopausal women is neither sensitive nor specific enough to warrant recommendations by the National Comprehensive Cancer Network^®^ (NCCN^®^), but it can be considered at the clinician’s discretion [59]. However, transvaginal ultrasound (TVUS) is not recommended as a screening tool for premenopausal women with LS, given the varying thickness of the endometrial stripe dependent on the phase of the menstrual cycle, making it difficult to distinguish physiologic changes from pathologic findings (Table 3) [59].

- Ovarian cancer

Women with LS should be counseled on the symptoms concerning OC, such as bloating, abdominal/pelvic pain, early satiety, change in bowel/bladder habits, and unintentional weight loss. There is currently no data to support the use of screening for OC in women with LS with either CA-125 or serial TVUSs. Nevertheless, CA-125 and pelvic ultrasound are recommended for preoperative planning for those patients scheduled to undergo risk-reducing surgery [59]. Table 3 highlights organ-specific surveillance, chemoprevention, and management recommendations for LS from different societies.

#### 5.4.2. Chemoprevention for LS-Associated Gynecologic Malignancies

- Endometrial cancer

Although not specifically studied in women with LS, hormonal therapies, including oral progestins, combined oral contraceptive pills, and the progesterone-containing intrauterine devices, may reduce EC risk for women with LS [59,125,127,128,129,130].

- Ovarian cancer

Similar to their use in the setting of EC, although not specifically studied in women with LS, combined oral contraceptives may reduce the risk of OC in this population [59].

#### 5.4.3. Risk-Reducing Surgery for LS-Associated Gynecologic Malignancies

- Endometrial cancer

A prophylactic total hysterectomy can be considered as a risk-reducing option for women with LS who have completed childbearing, as it has been shown to reduce the incidence of EC, but its impact on overall mortality remains uncertain. The timing of performing this procedure should be individualized based on patient comorbidities, family history, and the involved LS gene. In addition, guidelines regarding risk reduction recommendations vary between organizations. Recommendations for risk reduction strategies from five well-established professional organizations are highlighted in Table 3.

- Ovarian cancer

Bilateral salpingo-oophorectomy (BSO) may reduce the risk of OC in women with LS. The decision to proceed with BSO for risk reduction in women with LS should be individualized based on comorbidities, menopausal status, family history, completion of childbearing, and the mutated gene [59]. Similar to those for EC, risk reduction strategies for OC in patients with LS have been suggested, but these recommendations vary between organizations (Table 3). Patients should be counseled that premenopausal removal of the ovaries can have significant ramifications for a patient’s overall health, such as increased risk for osteoporosis, cardiovascular disease, and both sexual and cognitive dysfunction. Estrogen replacement should be considered in women who undergo risk-reducing BSO before the onset of menopause, although this intervention has not been specifically studied in women with LS [59]. Importantly, estrogen replacement has been shown to be protective against the development of CRC in the general population [131]. Given the health ramifications for risk-reducing BSO in premenopausal women, another consideration is performing opportunistic BSO for premenopausal women with LS.

#### 5.4.4. Oncologic Treatment Considerations for LS-Associated Gynecologic Malignancies

There are targeted treatment options for dMMR tumors for women with gynecologic malignancies and LS. The use of immunotherapy with checkpoint inhibitors such as durvalumab, dostarlimab, or pembrolizumab has been shown to have the greatest benefit in tumors that are dMMR, which is a key characteristic of Lynch-associated tumors. Either pembrolizumab or dostarlimab can be used with carboplatin/paclitaxel for women with advanced-stage/recurrent EC or as a single agent for dMMR recurrent EC [132]. Pembrolizumab is also an option for recurrent OC that is dMMR [133]. Lastly, durvalumab is also approved for combination carboplatin/paclitaxel for advanced-stage/recurrent EC that is dMMR [132].

### 5.5. Urothelial Cancer

Urothelial cancer (UC) in LS can arise in both the upper (renal pelvis, ureter) and lower (bladder) urinary tract. *MSH2* PVs confer the highest UC risk (Table 2). In a case–control study, individuals with LS had significantly higher rates of urinary tract cancer (UTC) than controls (4.1% vs. 1.2%; *p* < 0.0001), with distribution including bladder (42%), ureter/renal pelvis (39%), and kidney (31%). Risk was independently associated with male sex, increasing age, family history of UTC, and *MSH2/EPCAM* variants [134]. LS has a reported prevalence of 5.2% in upper tract urothelial carcinoma (UTUC) [135].

UC has demonstrated the highest LS carrier rate (37.5%) among MSI-H tumors [81]. dMMR rates in UTUC are comparable to those observed in CRC and EC, reinforcing the rationale for UTS [136]. Reflecting this, both the American Urological Association (AUA) and European Association of Urology (EAU) recommend universal MMR testing for patients with UTUC [137,138]. The 2023 EAU guidelines align with NCCN recommendations for germline testing in patients with UTUC but differ in age thresholds, recommending screening for those diagnosed before age 60, compared to age 50 in the NCCN Guidelines [59,138].

### 5.6. Biliary Tract Cancer

Individuals with LS, especially *MLH1* carriers, have an elevated risk of biliary tract cancer (BTC) (Table 2). In a retrospective study, 11 LS patients were identified with BTC, most of whom were male and presented symptomatically with advanced disease [139]. Similarly, Japanese studies have reported BTC in 4–6.5% of LS patients, predominantly involving *MLH1* [140,141]. All LS-associated BTCs tested were MSI-H, highlighting the utility of MSI testing in identifying LS [141].

Recent proposals have suggested BTC surveillance, particularly for *MLH1* carriers in high-incidence regions, using annual blood tests and imaging starting at age 40–50, or 5 years before the earliest family diagnosis [141]. Surveillance-detected cases were diagnosed at earlier stages with better outcomes, underscoring the potential value of structured screening for BTC in LS [141].

### 5.7. Pancreatic Cancer

Pancreatic cancer (PC) risk is increased in LS, particularly among *MLH1* carriers (Table 2). In a large prospective cohort of LS patients, the cumulative risk of PC by age 75 was highest for *MLH1* carriers (RR 7.8), while no increased risk was observed for *PMS2* carriers; however, data on *PMS2* remain limited [142].

In contrast, a recent analysis of 4626 PC cases identified 32 LS carriers, with *PMS2* among the most frequent (25%) [143]. These findings raise important questions about the surveillance for *PMS2* carriers, and current NCCN and International Cancer of the Pancreas Screening guidelines do not recommend PC surveillance for *PMS2* carriers [59,144]. Most tumors were pancreatic ductal adenocarcinomas, and 59% were MSI-H [143]. Median age at diagnosis was 68 years, with *MSH2* or *MLH1* carriers diagnosed at younger ages [143]. Immune checkpoint blockade showed a 92.9% disease control rate, including two complete responses in *MSH2* carriers [143].

### 5.8. Cutaneous Tumors

LS-associated skin tumors include SA, SC, and KA. The prevalence of LS among patients with SNs ranges from 18.8% to 33.3% [145,146], exceeding that observed in LS-associated CRC and EC.

dMMR is frequently observed in SC. One study reported abnormal IHC in 55% of sebaceous lesions and confirmed LS in 14% of those who underwent germline testing [147]. Another study found 32% of SCs were dMMR, with LS confirmed in 50% of tested individuals [148]. The LS UK Sebaceous Carcinoma group recommends universal MMR screening in SCs, as dMMR SC may be the initial manifestation of LS [63]. The NCCN Guidelines recommend considering a skin exam every 1–2 years, with the starting age for surveillance subject to individualization [59].

### 5.9. Brain Tumors

LS is associated with an increased risk of brain tumors, particularly glioblastomas. LS has been reported in 0.54% of unselected brain tumors and 0.41% of gliomas [149,150], with most LS-associated cases involving *IDH*-wildtype glioblastomas. The reported mean age at diagnosis was 31 years, and dMMR was more common in younger patients [150]. Based on these findings, screening for dMMR and LS is recommended for all patients diagnosed with *IDH*-wildtype glioblastomas before age 50 [150]. dMMR status in gliomas may confer resistance to temozolomide and increase responsiveness to immune checkpoint blockade [150,151]. Individuals with LS should be educated on the signs and symptoms of brain tumors and advised to promptly report any neurologic changes to their physicians [59].

### 5.10. Cancers with Emerging or Uncertain Associations with LS

#### 5.10.1. Prostate Cancer

The association between prostate cancer and LS remains under debate. The prevalence of LS identified through tumor testing in prostate cancer appears low, ranging from 0.29% to 0.8% across studies [81,152]. In contrast, the IMPACT study, a prospective international trial that followed 644 men with LS and 318 age-matched non-carriers, found that prostate cancer incidence was significantly higher in *MSH2* (4.3%) and *MSH6* (3.0%) carriers, with no cases observed in *MLH1* carriers [153]. Clinically significant prostate cancer occurred in 3.6% of *MSH2* carriers versus 0% in non-carriers, leading the study authors to recommend targeted PSA screening in men with *MSH2* and *MSH6* variants [153]. To date, no published consensus exists regarding prostate cancer surveillance in men with LS. The NCCN Guidelines recommend considering beginning shared decision-making about prostate cancer screening at age 40, with annual intervals [59].

#### 5.10.2. Breast Cancer

The association between LS and breast cancer (BC) remains unresolved. Breast cancers that develop in individuals with LS may exhibit genomic or histochemical features of LS, such as MSI-H or dMMR [59]. In a population-based analysis, Roberts et al. reported statistically significant BC increases for *MSH6* (SIR = 2.11; 95% CI, 1.56–2.86) and *PMS2* (SIR = 2.92; 95% CI, 2.17–3.92), while *MLH1* and *MSH2* were not significantly associated with BC [154]. By contrast, Harkness et al. (2015) found a higher cumulative BC risk to age 70 in *MLH1* carriers (18.6%; 95% CI, 11.3–25.9) versus both the UK general population (7.5–8%) and *MSH2* carriers (11.2%; 95% CI, 1.4–21.0; *p* = 0.014) [155]. Engel et al. observed an overall elevated BC risk (SIR = 1.9; 95% CI, 1.4–2.4) without gene-specific differences among *MLH1*, *MSH2*, and *MSH6* [156]. Several other studies reported no significant association between LS and BC [22,157,158]. Most recently, a large UK Biobank analysis found no increase in BC incidence among carriers of pathogenic *MLH1*, *MSH2*, *MSH6*, or *PMS2* variants [159], arguing against classifying BC as part of the LS tumor spectrum. Taken together, while some associations have been reported, the overall data do not consistently support an elevated breast cancer risk in LS. Further large studies are warranted.

#### 5.10.3. Sarcomas

The incidence of non-epithelial cancer, such as sarcoma, has been documented in individuals with LS. A study of nearly 1000 families with LS found that 5.7% of families had individuals with sarcoma (n = 58), with the majority in families with *MSH2*/*EPCAM* PVs [158]. A diverse range of sarcoma subtypes have been reported, such as osteosarcoma, undifferentiated pleomorphic sarcoma, leiomyosarcoma, liposarcoma, and rhabdomyosarcoma [160,161]. Typically, sarcomas in individuals with LS tend to be dMMR or MSI-H and may respond to immunotherapy like anti-programmed death receptor-1 (PD-1) treatments [162,163].

#### 5.10.4. Adrenocortical Carcinoma

Adrenocortical carcinoma (ACC) is a rare malignancy that has been found in individuals with LS [164,165]. Raymond et al. found that 3.4% of individuals with ACC may have LS, which is comparable to the prevalence among those with EC or CRC [166]. A study of patients in Catalonia found that 0.47% of individuals with LS had ACC, compared to an incidence of about 5.2 in 100,000 in the general population [167]. *MSH2* is the most common LS causative gene found in ACC [164,165,166,167]. While there has been increased recognition of ACC as an LS-associated malignancy, there is a substantial absence of data to support the efficacy of immunotherapy in patients with this tumor type. Much knowledge is needed to address this gap, particularly given that the standard treatment of such patients is cytotoxic chemotherapy, which is associated with significant toxicities and often results in dose reductions and modifications. Further, despite the association of ACC with LS and their shared affected mismatch repair genes, in their case report, Casey et al. describe the lack of efficacy of immune checkpoint inhibition in a patient with ACC, posing the question of whether the high levels of cortisol found in patients with cortisol-secreting ACC may sensitize or prevent an enhanced T cell response [168].

## 6. Differential Diagnoses

### 6.1. Hereditary Cancer Syndromes

Hereditary cancer syndromes that should be considered in the differential diagnosis of LS, including their associated colorectal and extracolonic features, are summarized in Table 4.

### 6.2. Sporadic Colorectal Cancers

Sporadic CRCs are more commonly diagnosed after age 50 and typically lack a strong family history. In such cases, the diagnosis of LS may be delayed, and the malignancy may be misattributed to lifestyle or environmental factors. To differentiate LS from sporadic CRC, obtaining a detailed three-generation family history is essential. Clinical red flags include early-onset CRC, multiple affected relatives on the same lineage, and LS-associated extracolonic malignancies. Individuals meeting clinical criteria, such as the Amsterdam II or revised Bethesda guidelines, should be referred for germline testing.

Intact MMR protein expression on IHC, especially in the absence of a strong family history, often suggests a sporadic etiology. Most sporadic CRCs are MSI-low or MSS; however, some can also exhibit MSI-H status. Tumor profiling via NGS can be informative, especially in metastatic disease, by assessing MSI status and detecting pathogenic single-nucleotide variants (SNVs) or structural variants (SVs) in MMR genes. Because PVs/LPVs in MMR genes identified in tumor tissue may represent either somatic or germline alterations, their detection should be followed by confirmatory germline testing in accordance with current clinical guidelines.

**Figure 4:**
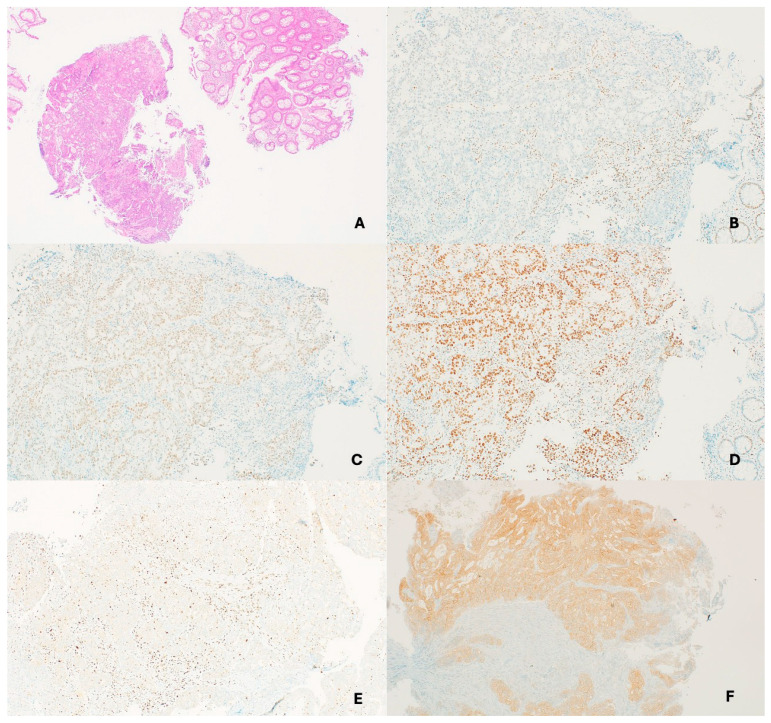
(**A**) H&E low-power view of colon adenocarcinoma (4×); (**B**) Loss of *MLH1* protein expression (10×); (**C**) Retained *MSH2* protein expression (10×); (**D**) Retained *MSH6* protein expression (10×); (**E**) Loss of *PMS2* protein expression (10×); and (**F**) Immunoreactive tumor cells against *BRAF* V600E immunohistochemical stain (4×), confirming the sporadic nature of this tumor.

**Figure 5:**
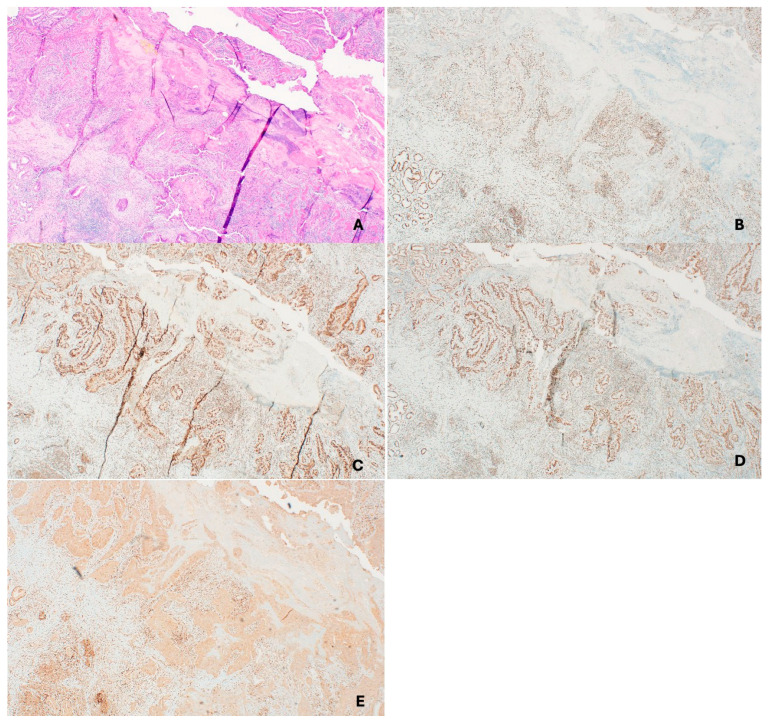
(**A**) H&E low power (4×) view of a perihilar cholangiocarcinoma in a 75-year-old male with significant past medical history including colorectal adenocarcinoma, leiomyosarcoma, squamous cell carcinoma of the vocal cords, and skin squamous cell carcinoma; (**B**) Loss of *MLH1* protein expression (10×); (**C**) Retained *MSH2* protein expression (10×); (**D**) Retained *MSH6* protein expression (10×); and (**E**) Loss of PMS2 protein expression (10×). Next-generation sequencing performed on tumor material demonstrated an *MLH1* mutation and microsatellite-stable status. Germline mutation analysis confirmed the presence of the *MLH1* mutation, thus supporting the diagnosis of LS.

Somatic mutations in *KRAS* or *BRAF*, particularly the *BRAF* V600E variant, are more commonly seen in sporadic CRCs and help distinguish them from LS. If *BRAF* IHC is unavailable, *MLH1* promoter hypermethylation studies can be performed on the tumor material. A positive result for *MLH1* promoter methylation or *BRAF* mutation supports a sporadic tumor. Conversely, the absence of both findings should prompt germline testing for LS (Figure 2, Figure 4 and Figure 5) [169].

## 7. The Utilization of Immune-Mediating Agents in the Management of Individuals with Lynch Syndrome

The fundamental principles of managing individuals with LS include preventing cancer development through surveillance and eliminating premalignant lesions. They also include treating existing cancer by resecting localized tumors or using various modalities to eradicate malignancy or control disease progression in advanced or metastatic settings. As a substantial proportion of cancers that develop in the setting of LS exhibit dMMR or MSI-H, the treatment algorithms for their management largely follow those recommended for the therapy of sporadically developed tumors with these same characteristics. These shared features result in a rich immune microenvironment, allowing consideration of immune-mediating agents, such as ICIs, as therapeutic options. While dMMR/MSI-H cancers that develop sporadically or arise from a genetic predisposition are subsets of malignancies that have both been demonstrated to possess immunogenic properties, profound distinctions in humoral and T cell responses exist between the precursor lesions that develop in each setting [170,171]. Further, research has shown that antibody responses against frameshift peptides (FSPs) are endogenously induced in healthy individuals with LS-defining germline gene variants who have no history of tumor development, albeit to a lesser extent than in those who have developed LS-CRC [171,172]. Similarly, FSP-specific effector T cell responses have been detected in the peripheral blood of healthy LS patients with no history of cancer, as well as in those with LS-CRC [108,170,173,174]. These findings support the evaluation of immunoprevention and immune interception in those with LS, concepts that continue to garner interest among those committed to improving outcomes in this population.

### 7.1. Immune Checkpoints and the Use of Immune Checkpoint Blockade in the Treatment of LS-Associated Cancer

Immune checkpoints are proteins that exist on the surface of immune cells that play a critical role in the regulation of immune self-tolerance [175]. These molecules, classified as either inhibitory or stimulatory, aid in the avoidance of indiscriminatory attack on cells by the immune system, thus contributing to the maintenance of immune homeostasis. In recent years, a greater understanding of the function of these proteins through their interaction with ligands present on tumor cells has revolutionized the field of oncology by introducing another modality for use in the treatment of certain types of cancer. Through their utilization as monotherapy or in conjunction with chemotherapy and/or radiotherapy, ICIs have been demonstrated to impart targeted, durable responses, with these effects appearing to be most pronounced when used in the treatment of tumors with exceptionally high mutation rates (characterized as hypermutator phenotypes; e.g., melanoma, endometrial, cervical, gastric, and colorectal cancers).

In early 2017, four ICIs were granted approval by the U.S. FDA for their use in the treatment of certain cancers. Notably, pembrolizumab (a PD-1-blocking antibody) was approved for use in the management of patients with previously treated unresectable or metastatic dMMR or MSI-H solid tumors and for use, specifically, in individuals with dMMR/MSI-H CRC whose cancers progressed following treatment with a fluoropyrimidine, oxaliplatin, and irinotecan. These approvals were based on the results of five uncontrolled, open-label, multi-cohort, multicenter, single-arm trials that collectively assessed efficacy in 149 patients treated with pembrolizumab for MSI-H or dMMR cancers, of which 90 had CRC [176,177]. Subsequently, based on promising survival data from the KEYNOTE-177 (NCT02563002) study, pembrolizumab was granted FDA approval for its use in the first-line treatment of individuals with unresectable or metastatic MSI-H or dMMR CRC [178]. The PD-1 inhibitor nivolumab also received FDA approval as single-agent therapy for the treatment of patients with MSI-H or dMMR CRC that has progressed following treatment with a fluoropyrimidine, oxaliplatin, and irinotecan, or in combination with ipilimumab (an anti-cytotoxic T-lymphocyte antigen 4 [CTLA-4] antibody) as first-line therapy in patients with unresectable or metastatic MSI-H or dMMR CRC based on the CheckMate-142 (NCT02060188) and CheckMate-8HW (NCT04008030) studies, respectively [179,180].

The FDA has also approved several ICIs, in combination with chemotherapy or as single agents, for the treatment of EC, the development of which individuals with LS are also known to be at increased risk, and of which a proportion exhibit dMMR and/or MSI-H. The combination of durvalumab (a programmed death-ligand 1 [PD-L1] blocking antibody) with carboplatin and paclitaxel, followed by single-agent durvalumab, is approved for the treatment of patients with primary advanced or recurrent dMMR EC. This approval was based on findings from the DUO-E study (NCT04269200), a randomized, multicenter, double-blind, placebo-controlled trial in patients with primary advanced or recurrent EC, which included tumor MMR status as a stratification factor [181]. Other indications utilizing the combinations of pembrolizumab with carboplatin and paclitaxel, and dostarlimab with these chemotherapeutic agents, have also been approved, but with the noteworthy distinction of their use in advanced or recurrent EC, regardless of MMR status. These MMR-independent approvals indicate a revolutionary shift from dependence on specific biomarker expression or molecular status (such as dMMR or MSI-H) to the consideration that the addition of these agents to chemotherapy and targeted agents in the treatment of tumors not considered highly immunogenic may overcome the inherent resistance to immunotherapy that these types of tumors typically exhibit. In addition, pembrolizumab is also approved for use in combination with lenvatinib (a multiple receptor tyrosine kinase inhibitor) in the treatment of patients with advanced EC that is pMMR or MSS whose disease has progressed on prior systemic therapy and who are not candidates for curative surgery or radiation. The synergistic mechanism that results from the combination of lenvatinib and pembrolizumab relies on the enhancement of immune cell infiltration and activation through the modification of the TME (check where the abbreviation first occurs for “tumor microenvironment”). The inhibition of VEGF-induced neovascularization by lenvatinib facilitates the infiltration of immune cells into the tumor, decreases the presence of suppressive TAMs and Tregs (check when abbreviation first occurs), enhances dendritic cell activity, and increases the release of pro-inflammatory cytokines [182]. Pembrolizumab restores the function of exhausted T-cells, thereby stimulating immune response and blocking tumor evasion strategies [183]. These three approvals were based on findings resulting from KEYNOTE-868/NRG-GY018 (NCT03914612), RUBY (NCT03981796), and Study 309/KEYNOTE-775 (NCT03517449) studies [184,185,186]. These studies emphasize the potential impact that the addition of an ICIs to conventional chemotherapy or to targeted therapy may have on clinical outcomes for those with EC. Regarding options for monotherapy, both pembrolizumab and dostarlimab (a PD-1 inhibitor) have each been granted approval for their use as single agents in previously treated patients with advanced dMMR (or MSI-H in the case of pembrolizumab) EC who are not candidates for curative surgery or radiation, based on the results of clinical studies demonstrating improved progression-free survival (PFS) with use of these agents in this setting [187,188].

A vast number of trials seeking to evaluate the effect of immunotherapy with or without the concurrent use of chemotherapy and/or targeted agents on active disease, both in the first-line setting, as well as use in subsequent therapy in previously treated individuals are ongoing; in addition, numerous studies are also attempting to decipher the optimal sequence of treatment of these agents, as well as determine efficacy of various timing of therapy (e.g., neoadjuvant, peri-operative, adjuvant treatment). While the findings resulting from all of these investigations will certainly add to our knowledge regarding the treatment of existing malignancies, they will undoubtedly also serve to inform the field regarding the optimization of the use of immunotherapy in the preventive setting. This understanding will be particularly critical for those at risk of developing tumors with features rendering them “hot” or potentially more responsive to this type of therapy, such as the neoplasms known to occur in individuals with LS.

### 7.2. Vaccines for Immunoprevention in LS

Like sporadic MSI-H tumors, MSI-H LS-associated malignancies exhibit impaired MMR. This malfunctioning process results in the generation of altered carboxy-terminal peptide sequences secondary to MSI-induced shifts in the translational reading frame [174]. These FSPs, or neoantigens, are tumor-specific and highly immunogenic; in contrast, tumor-associated antigens are present on both tumor and normal cells and exhibit lower immunogenicity than their neoantigen counterparts. Neoantigens can evoke several types of immune responses; however, a profound distinction exists in that the provocation of such immune responses occurs even prior to the development of clinical disease. Premalignant lesions in LS, such as adenomatous polyps, arise in a robust immune microenvironment, with T cell infiltration and an upregulation of immune-related genes as prominent features. For example, mutations in *BAX2* and *TGFBR2* occur early and are found in over half of LS adenomas; further, mutations continue to accumulate during the progression to high-grade adenomas [109,189].

As these lesions progress to advanced adenomas, there is also an increase in the neoantigen burden and markers of immune tolerance [190]. The upregulation of immune-related genes, however, has been demonstrated to be independent of mutational rate and neoantigen load, lending support to the concept of implementing immunoprevention as a prophylactic intervention, as this observation suggests that immune activation is an early occurrence in LS-associated tumorigenesis, rather than the consequence of the accumulation of somatic mutations [191,192].

Risk reduction has traditionally and heavily relied on intense surveillance strategies and procedural intervention. However, our growing understanding of the immune processes and responses that occur in individuals with LS has propelled vaccine-based work in the pursuit of an alternative strategy for disease prevention in this population. Both neoantigens and tumor-associated antigens have been proposed as potential targets in this approach. Findings from an open-label single-arm phase I/IIa clinical study (NCT01461148) evaluating the safety and immunogenicity of a vaccine generated from FSP neoantigens derived from mutant *AIM2*, *HT001*, and *TAF1B* genes in patients with a history of LS-associated or sporadic dMMR/MSI CRCs demonstrated that vaccination was safe, well-tolerated, and induced both cellular and humoral immune responses in all vaccinated patients [173]. Further, among previously treated patients with measurable disease at the time of vaccination, several had stable disease as their best overall response at follow-up. A similar effort to assess safety, tolerability, and immune response to vaccination of frameshift-derived neoantigens was undertaken by investigators in another phase I/II clinical trial (NCT01885702). However, in this study, the administered product was monocyte-derived dendritic cells loaded with frameshift-derived neoantigens of *TGFBR2* and caspase-5, in addition to tumor-associated peptide carcinoembryonic antigen (CEA) [193]. Participants included healthy individuals with LS, as well as those with LS who had previously developed CRC, but who had undergone surgical resection of their cancers. Vaccination was found to be safe and well-tolerated, and to induce antigen-specific T cell responses, including T cell cytotoxicity against tumor cells presenting the TGFBR2 neoantigen, in several patients, with production of multiple cytokines and high levels of IFN-gamma detected in the peripheral blood of 87% of vaccinated patients [193].

Another study (NCT05078866) has found that Nous-209, an “off-the shelf” vaccine encoding 209 neoantigens shared across both sporadic and hereditary MSI tumors based on a heterologous prime/boost regimen composed of the Great Ape Adenovirus GAd20-209-FSP used for priming and Modified Vaccinia virus Ankara MVA-209-FSP used for boosting, was well-tolerated and associated with robust immunogenicity against FSP neoantigens in healthy LS carriers [194,195]. Specifically, vaccination with Nous-209 elicited T cell immune response against recurrent FSPs previously identified in MSI tumors in all evaluable patients. A multicenter phase 2 randomized clinical trial evaluating Nous-209 (NCT04041310) is currently ongoing and includes a 2:1 randomized cohort of previously untreated patients with metastatic MSI CRC receiving Nous-209 plus pembrolizumab versus pembrolizumab alone, and a single-arm cohort of patients with metastatic MSI CRC who have become refractory to prior anti-PD-1 therapy receiving Nous-209 plus pembrolizumab. Efficacy will be assessed, with overall response rate, disease control rate, overall survival, and PFS among the clinical parameters to be evaluated; in addition, immunogenicity will be determined. Results from these studies are anticipated to inform strategies of disease interception at the premalignant stage, as well as provide additional insight into overcoming resistance to immune checkpoint inhibition and the effect of a dual approach of vaccine in combination with checkpoint blockade on active disease.

Carcinoembryonic antigen and mucin 1 (MUC1) are cancer antigens that are known to be expressed by the malignancies for which patients with LS are at risk [196,197,198,199]. These antigens have been shown to elicit immune responses in individuals with advanced adenomas and in those with late-stage colorectal adenocarcinomas, supporting their evaluation as candidate targets for vaccine prevention in individuals with LS [200,201,202,203,204]. An ongoing multicenter phase IIb clinical trial (NCT05419011) aims to evaluate whether the combination of trivalent adenovirus-5 (Tri-Ad5) vaccines and the IL-15 superagonist nogapendekin alfa inbakicept (N-803) can reduce the incidence of colorectal neoplasms in patients with LS. The Tri-Ad5 vaccine is a combination of three vaccines targeting the tumor-associated antigens CEA, MUC1, and brachyury, while N-803 is a complex of IL-15/IL-15Rα with greater stability, potency, and bioavailability than soluble IL-15 [201,203,205,206,207]. Further, N-803 is known to preferentially stimulate activation, proliferation, survival, and cytotoxicity of NK and CD8+ T cells, including memory CD8+ T cells [206,207]. In addition to safety, tolerability, and immunogenicity, the cumulative incidence of colorectal neoplasms on follow-up colonoscopies will be determined. Importantly, the effect of this regimen on the incidence of LS-related extracolonic cancers will be assessed; additionally, it is approved for intravesical use in combination with BCG in the treatment of BCG by investigators. Notably, the IL-15 agonist, nogapendekin alfa inbakicept-pmln, is unresponsive in non-muscle invasive bladder cancer.

## 8. Genetic Counseling

### 8.1. Inheritance

LS is inherited in an AD pattern. Thus, anyone with a heterozygous PV/LPV in *MLH1*, *MSH2*, *MSH6*, *PMS2*, or *EPCAM* would have a molecular diagnosis of LS and would be considered at elevated risk for LS-associated cancers [37]. First-degree relatives (children, full siblings, and parents) of individuals with LS would therefore have up to a 50% chance of also having the same PV/LPV.

### 8.2. Genetic Information Nondiscrimination Act (GINA) and Related Legal and Ethical Considerations

An important consideration during the pretest counseling process is the possibility of genetic discrimination. Within the U.S., the Genetic Information Nondiscrimination Act (GINA) is a federal law passed in 2008 meant to help protect patients from facing discrimination based on genetic testing results [208]. According to GINA, patients cannot face discrimination from their health insurers or employers based on the results of a genetic test [208]. Though GINA introduces essential protections for patients, it has notable limitations [209]. Not all employers are covered by GINA; hence, these organizations (a notable example being the U.S. military) can use genetic testing results to an extent when making employment decisions [210]. Only health insurance is covered by GINA, not life, long-term care, or disability insurance [208,209,210,211]. Medical providers are not always familiar with the scope and limitations of GINA. Therefore, it is critical that clinicians ordering genetic testing for LS review these regulations and discuss them with their patients before they initiate testing.

### 8.3. Family Planning and Preimplantation Genetic Testing

Family planning for individuals with LS can be critical in multiple ways. Given that management for LS ultimately includes a recommendation for total hysterectomy, typically with BSO, timing of pregnancy can be very important, and therefore, people with LS might consider pregnancy and family planning earlier in life if they desire biological children without the use of a surrogate. In addition, there can be other aspects of family planning to consider, such as the desire not to pass on the same PV to their children. This can be achieved using preimplantation genetic testing for monogenic disorders, or PGT-M. With this, people can use in vitro fertilization to test developing embryos for the familial LS mutation; then, they can implant embryos that do not carry it, effectively preventing the transmission of LS to the next generation. Likewise, this can prevent the risk of CMMRD in potential pregnancies. People with LS should be informed of these services as another option for family planning.

### 8.4. Genetic Testing Considerations in Special Populations: Pediatric and Military Populations

Genetic testing for hereditary cancers is generally recommended only for adults, as the majority of individuals will not experience an impact on their health management until adulthood [57,212]. However, the genes associated with LS introduce two important nuances to this principle. First, biallelic mutations in Lynch-related genes can cause CMMRD, which involves the risk of childhood cancer [213]. Second, some families with particularly aggressive histories of cancer associated with LS may benefit from pediatric testing, as it may impact a child’s screening before age 18 [57]. For instance, if the youngest age of CRC diagnosis in a family with an *MLH1* mutation is 21 years old, children at risk of inheriting this mutation may benefit from testing before the age of 18, such that they can begin recommended screening as necessary [57]. Such situations pose a unique counseling scenario requiring specialized psychosocial approaches and testing considerations. It is important that providers engage with genetic clinical services in such scenarios to ensure pediatric patients are offered appropriate counseling and testing [214]. This is an area of particular future interest in the growing era of genomic testing, as more children are being offered exome and genome testing that could reveal secondary findings of adult-onset conditions like LS [89,215], and future research should look into counseling best practices in these scenarios.

Another unique patient population comprises active-duty service members (ADSM) of the U.S. Armed Forces and their families. Having a personal diagnosis of LS is considered a disqualifying condition under the U.S. Department of Defense’s (DoD) Medical Standards for retention of U.S. military service members [216], and having a personal or family history of LS is considered a disqualifying condition for appointment, enlistment, or induction into the U.S military service [217]. The current extent of how widely these standards are applied, specifically to the active-duty population with personal/family histories of LS, is unknown, but such statutes may present a barrier to ADSM who consent to undergo testing that may potentially reveal life-saving information if their employment and financial benefits could be terminated based on positive genetic test results. It is important to note that these populations are not disproportionately affected by LS; rather, they present unique considerations for genetic testing and counseling when LS is identified or suspected.

## 9. Significance of Lynch Syndrome in Cancer Genetics: Knowledge Gaps and Future Directions

LS is significant in medical genetics as it represents one of the most common inherited cancer syndromes. Thanks to decades of work in LS, the field of cancer genetics has benefited from the knowledge obtained through research. LS not only teaches us about the role of genetics in cancer development, but it also teaches us about genetic concepts such as autosomal dominant and recessive patterns of inheritance, penetrance, expressivity, and genetic heterogeneity, among others, as highlighted in the description of LS.

Despite remarkable progress in understanding LS, several critical gaps remain. There is a major need to improve the rate of germline testing among patients with dMMR tumors, as undertesting continues to limit the timely identification of affected families. Advances in molecular technologies are needed to improve the detection of mosaic LS cases and complex structural variants that may be missed by conventional testing.

Surveillance strategies for extracolonic cancers still lack international consensus (Table 3). There is a growing emphasis on genotype-specific surveillance and prevention strategies, highlighting the importance of precision oncology in LS care. Preventive efforts should expand beyond colonoscopy and prophylactic surgery. Non-surgical risk reduction strategies, including chemoprevention and hormonal interventions, warrant further investigation. Further research is essential to define optimal management for patients with LLS and FCCTX.

Recent therapeutic innovation continues to evolve with the discovery of novel therapies targeting LS-associated tumors and the development of vaccines for the prevention of tumors, both of which rely on genetic information as a foundation for their development. Immunotherapy has revolutionized the treatment of advanced LS-associated cancers, while ongoing early-phase trials of FSP vaccines show promise for both treatment and prevention. Future directions should focus on refining individualized surveillance, expanding access to germline testing, and integrating genomic data into clinical decision-making to promote patient-centered approaches in the management of LS.

## 10. Conclusions

LS is the most common hereditary AD CRC syndrome, arising from GPVs in the DNA MMR genes MLH1, MSH2, MSH6, and PMS2, and from deletions in *EPCAM*. First described by Aldred Scott Warthin in 1913 and later defined by Henry T. Lynch in 1966 as “cancer family syndrome,” LS was formally renamed in the 1980s and has since become a model for hereditary cancer predisposition. The hallmark biology, dMMR leading to MSI, underpins its markedly increased risk of colorectal and extracolonic cancers, including endometrial, ovarian, gastric, and urothelial malignancies. Importantly, cancer risk is not uniform: carriers of *MLH1* and *MSH2* variants generally face higher lifetime risk and penetrance than those with *MSH6* or *PMS2* variants, enabling gene-specific counseling and surveillance recommendations.

Accurate and timely diagnosis is central to LS management. Integration of germline genetic testing, tumor-based assays (e.g., PCR, NGS, or IHC), and family history evaluation enables distinction from sporadic cancers and guides clinical decision-making. Once identified, cascade testing in at-risk relatives extends the benefits of surveillance and prevention to entire families, amplifying the clinical impact of diagnosis. Surveillance remains the cornerstone of care, with guideline-directed colonoscopy screening, gynecologic evaluation, and emerging risk-reduction interventions such as prophylactic hysterectomy and chemoprevention with aspirin. These strategies continue to improve survival and reduce morbidity, particularly when tailored to individual genetic profiles.

Recent advances have transformed the therapeutic landscape. The immunogenicity of MSI-H tumors has made immunotherapy the standard of care for advanced LS-related cancers. Checkpoint inhibitors such as pembrolizumab and the combination of nivolumab with ipilimumab demonstrate durable responses and improved tolerability compared with conventional chemotherapy. Beyond checkpoint blockade, novel approaches such as neoantigen-based vaccines hold promise for recurrence prevention and represent the next frontier in precision oncology.

Despite these advances, important challenges remain. Barriers to universal genetic testing, disparities in access to specialized care, and variable uptake of preventive strategies limit the full impact of LS diagnosis. Special considerations for genetic testing are also warranted for pediatric populations and military service members, where unique psychosocial and logistical factors may affect care. Furthermore, while GINA offers some protections, its limitations necessitate continued advocacy to ensure comprehensive safeguarding of patients and families.

Initiatives such as the International Mismatch Repair Consortium foster harmonization of surveillance protocols, encourage data sharing, and promote equitable implementation of precision medicine. Ultimately, the evolving molecular understanding of LS continues to shape surveillance, prevention, and therapeutic innovations. The overarching goal remains clear: to improve survival and quality of life for affected individuals and their families through multidisciplinary, lifelong care that integrates gastroenterology, oncology, genetics, gynecology, and pathology.

## Figures and Tables

**Table 1:** Comparison of Lynch Syndrome risk prediction models [83,84,85].

Comparison of Lynch Syndrome Risk Prediction Models			
Feature	PREMM5	MMRpredict	MMRPro
**MMR genes included**	*MLH1*, *MSH2*, *MSH6*, *PMS2*, *EPCAM*	*MLH1*, *MSH2*, *MSH6*	*MLH1*, *MSH2*, *MSH6*
**Methodology**	Polytomous logistic regression	Multivariate logistic regression	Mendelian and Bayesian models
**Clinical data utilized**	Personal and family history of LS-associated cancers	Personal history of CRC and family history of CRC and/or EC ^a^	Personal and family history of CRC and EC, age, molecular testing results
**Threshold for germline testing**	≥5% (or ≥2.5% if a personal history of CRC and/or EC)	≥5%	≥5%
**Online access** **(accessed on 2 November 2025)**	https://premm.dfci.harvard.edu	https://webapps.igc.ed.ac.uk/world/research/hnpccpredict/	https://projects.iq.harvard.edu/bayesmendel/mmrpro

^a^ MMRpredict model was developed from a population with colorectal cancer diagnosed under the age of 55 years. Created in BioRender. Eroglu S. (2025). https://BioRender.com/e8kkxmx (accessed on 2 November 2025).

**Table 2:** Cancer risks by MMR gene in individuals with Lynch Syndrome through age 80 years compared to the general population.

Cancer Site	Lifetime Cumulative Risk in the General Population	Cumulative Risk Through Age 80 and Estimated Average Age of Presentation			
		*MLH1*	*MSH2* and *EPCAM*	*MSH6*	*PMS2*
**Colorectal**	4%	46–61% 44 y	33–52% 44 y	10–44% 42–69 y	8.7–20% 61–66 y
**Endometrial**	3.1%	34–54% 49 y	21–57% 47–48 y	16–49% 53y–55y	13–26% 49–50 y
**Ovarian**	1.1%	4–20% 46 y	8–38% 43 y	≤1–13% 46 y	According to NCCN guidelines, it is unclear whether *PMS2* LS carriers have increased risk for other LS-associated cancers compared to the general population and data are insufficient to provide cancer risk estimates beyond those for CRC and EC.
**Gastric**	0.8%	5–7% 52 y	0.2–9% 52 y	≤1–7.9% *	
**Small bowel**	0.3%	0.4–11% 47 y	1.1–10% 48 y	≤1–4% 54 y	
**Pancreas**	1.7%	6.2% NA	0.5–1.6% NA	1.4–1.6% NA	
**Biliary tract**	NA	1.9–3.7% 50 y	0.02–1.7% 57 y	0.2–≤1% NA	
**Renal pelvis and/or ureter**	1.8%	0.2–5% 59–60 y	2.2–28% 54–61 y	0.7–5.5% 65–69 y	
**Bladder**	2.2%	2–7% 59 y	4.4–12.8% 59 y	1.0–8.2% 71 y	
**Prostate**	12.8%	4.4–13.8% 63 y	3.9–23.8% 59–63 y	2.5–11.6% 63 y	
**Brain**	0.6%	0.7–1.7% NA	2.5–7.7% NA	0.8–1.8% 43–54 y	

Abbreviations: NA, not available; CRC, colorectal cancer; EC, endometrial cancer. * Two cases reported at ages 45 and 81. Adapted with permission from the NCCN Clinical Practice Guidelines in Oncology (NCCN Guidelines^®^) for Genetic/Familial High-Risk Assessment: Colorectal, Endometrial, and Gastric V.1.2025. © 2025 National Comprehensive Cancer Network, Inc. All rights reserved. The NCCN Guidelines^®^ and illustrations herein may not be reproduced in any form for any purpose without the express written permission of NCCN. To view the most recent and complete version of the guideline, go online to NCCN.org. The NCCN Guidelines are a work in progress that may be refined as often as new significant data becomes available. NCCN makes no warranties of any kind whatsoever regarding their content, use, or application and disclaims any responsibility for their application or use in any way. Created in BioRender. Eroglu S. (2025). https://BioRender.com/tsl1uuf (accessed on 2 November 2025).

**Table 3:** Organ-specific surveillance, chemoprevention, and risk reduction recommendations for Lynch Syndrome: A comparative review of clinical guidelines [91,92,93,94].

	NCCN Version 1.2025	EHTG/ESCP 2021	JSCCR 2020	ESMO 2019	ASCRS 2016
**Colorectal Cancer**	*MLH1*, *MSH2*, and *EPCAM*: Colonoscopy at age 20–25 or 2–5 y before earliest CRC if it is diagnosed <25 y and repeat every 1–2 y. *MSH6* and *PMS2*: Colonoscopy at age 30–35 or 2–5 y before earliest CRC if diagnosed < 30 y and repeat every 1–3 y.	Colonoscopy *MLH1* and *MSH2*: Start at age 25; every 2–3 y. *MSH6*: Start at age 35; every 2–3 y. *PMS2*: Start at age 35 y; every 5 y.	Colonoscopy at age 20–25 and repeat every 1–2 y; start 2–5 y earlier if CRC occurred before age 25 in the family. For *MSH6*, consider starting at 30 y or 10 y before the youngest CRC case. For *PMS2*, consider starting at 35 y.	*MLH1* and *MSH2:* Colonoscopy at age 25 or 5 y prior to the earliest CRC if it is diagnosed before age 25, and repeat every 1–2 y *MSH6* and *PMS2:* Colonoscopy at age 35 or 5 y prior to the earliest CRC and repeat every 1–2 y.	Recommend colonoscopy every 1–2 y starting at age 20–25, or 2–5 y before the youngest CRC diagnosis in the family if before age 25. Consider annual colonoscopy. For *MSH6*, consider starting at age 30; for *PMS2*, at age 35.
**Aspirin Chemoprevention**	The Panel recommends all individuals with LS who have a risk for future CRC consider using daily aspirin to reduce their future risk of CRC. Dose, duration, and patient-specific risks should be individually assessed.	Patients should be advised that daily aspirin reduces CRC risk. Recommend at least 75–100mg daily, with higher doses for individuals with above-average body mass.	Not using aspirin for cancer prevention is weakly recommended at this time.	Consider aspirin for cancer prevention; optimal dose has not been established.	Consider aspirin for cancer prevention after discussion of risks, benefits, and uncertainties of treatment.
**Gastric and Small Bowel Cancer**	*MLH1*, *MSH2*, *MSH6*, and *EPCAM:* EGD at age 30–40 and repeat every 2–4 y. Starting prior to 30 y and/or interval <2 y may be considered based on family history or endoscopic findings. Consider extended duodenal exam. Random biopsy of the proximal and distal stomach should at minimum be performed on the initial procedure to assess for *H. pylori*, autoimmune gastritis, and intestinal metaplasia. Individuals not undergoing upper endoscopic surveillance should have one-time noninvasive testing for *H. pylori*	There is no demonstrated benefit of surveillance for incidence or survival of gastric or small bowel cancer. Consensus was not achieved for the statement: “Surveillance for other cancers (other than CRC, EC, and OC) should not be offered”.	Consider upper gastrointestinal endoscopy every 1–3 y starting at age 30–35 in individuals at high-risk or with a family history of gastric or duodenal cancer. Screen for *H. pylori* infection at age 30–35, and treat if infection is present.	Consider upper gastrointestinal endoscopy every 1–3 y starting at age 30–35 in individuals with a family history of gastric cancer or in high-incidence regions. Consider testing and treating *H. pylori.* Routine surveillance of the small bowel in LS has a high rate of false-positive findings and it is not considered to be cost-efficient.	Consider upper gastrointestinal endocscopy with antral biopsy starting at age 30–35 and repeat every 2–3 y based on individual risk. Treat *H. pylori* if detected. Routine screening of the small intestine is not recommended.
**Pancreatic Cancer**	Consider annual screening starting at age 50, or 10 y earlier than the youngest PC diagnosis in the family, using contrast-enhanced MRI/MRCP and/or EUS in *MLH1*, *MSH2*, *MSH6*, and *EPCAM* carriers with ≥1 first- or second-degree relative affected.	There is no demonstrated benefit of surveillance for incidence or survival of pancreatic cancer.	NA	Conisder annual MRI and/or EUS in individuals with a first-degree relative with pancreatic cancer.	Routine screening of the pancreas is not recommended.
**Endometrial Cancer**	Educate patients on EC symptoms. Consider endometrial biopsy every 1–2 y starting at age 30–35. TVUS may be considered in postmenopausal patients. Risk-reducing hysterectomy (with BSO) may be considered starting at age 40 for *MLH1* and *MSH2*, and at age 50 for *PMS2* carriers. For *MSH6* carriers, hysterectomy with bilateral salpingectomy starting at age 40 with delayed bilateral oophorectomy starting at age 50 may be considered.	Consider annual review to discuss red flag symptoms for EC/OC from age 25. Invasive gynecologic surveillance is not recommended. Offer risk-reducing total hysterectomy and BSO from age 35–40 after childbearing for *MLH1, MSH2,* and *MSH6* carriers.	Consider annual endometrial biopsy from age 30–35; cytology may be used at the physician’s discretion. TVUS is not recommended for surveillance, especially in premenopausal women. Risk-reducing surgery should be considered based on childbearing status and comorbidities.	Recommend annual gynecological exam, TVUS, and endometrial biopsy starting at age 30–35. Prophylactic hysterectomy with bilateral oophorectomy may be considered after childbearing or in postmenopausal individuals.	Screening should be offered to women by pelvic examination and endometrial sampling annually starting at age 30–35. Hysterectomy and BSO should be recommended after childbearing or at age 40.
**Ovarian Cancer**	*MLH1*, *MSH2*, *MSH6*, and *EPCAM:* Routine screening is not recommended. Salpingectomy may be considered in premenopausal patients who are not ready for oophorectomy. Oral contraceptive pills and progestin intrauterine systems may also be considered for EC and OC.		Consider transvaginal ultrasound and CA-125 annually starting at age 30–35 at the physician’s discretion. Patients should be advised to promptly report symptoms suggestive of OC.	Recommend annual gynecological examination with TVUS and CA-125 testing starting at age 30–35.	Screening should be offered to women by TVUS annually starting at age 30–35.
**Urothelial Cancer**	MLH1, MSH2, MSH6, and EPCAM: Consider surveillance in selected individuals (e.g., family history of urothelial cancer) with annual urinalysis starting at age 30–35.	There is no demonstrated benefit of surveillance for incidence or survival of urinary tract cancers.	Consider annual urinalysis (or urine cytology) starting at age 30–35 in individuals with PVs in *MSH2* or a family history of UC.	Consider surveillance in individuals with a family history of urothelial cancer. Surveillance is recommneded only under a research protocol.	Consider annual urinalysis starting at age 30–35.

Abbreviations: NCCN, National Comprehensive Cancer Network; EHTG, European Hereditary Tumor Group; ESCP, European Society of Coloproctology; JSCCR, Japanese Society for Cancer of the Colon and Rectum; ESMO, European Society for Medical Oncology; ASCRS, American Society of Colon and Rectal Surgeons; CRC, colorectal cancer; EGD, esophagogastroduodenoscopy; PC, pancreatic cancer; MRI, magnetic resonance imaging; MRCP, magnetic resonance cholangiopancreatography; EUS, endoscopic ultrasonography; TVUS, transvaginal ultrasound; BSO, bilateral salpingo-oophorectomy; NA, not available. As premature menopause due to oophorectomy can cause detriments to bone health, cardiovascular health, and generalized quality of life, estrogen replacement therapy should be considered [59]. For patients of reproductive age, advise about options for prenatal diagnosis and assisted reproduction, including preimplantation genetic testing. Discussion should include known risks, limitations, and benefits of these technologies [59]. Adapted with permission from the NCCN Clinical Practice Guidelines in Oncology (NCCN Guidelines^®^) for Genetic/Familial High-Risk Assessment: Colorectal, Endometrial, and Gastric V.1.2025. © 2025 National Comprehensive Cancer Network, Inc. All rights reserved. The NCCN Guidelines^®^ and illustrations herein may not be reproduced in any form for any purpose without the express written permission of NCCN. To view the most recent and complete version of the guideline, go online to NCCN.org. The NCCN Guidelines are a work in progress that may be refined as often as new significant data becomes available. NCCN makes no warranties of any kind whatsoever regarding their content, use, or application, and disclaims any responsibility for their application or use in any way. Created in BioRender. Eroglu, S. (2025) https://BioRender.com/lsea5x5 (accessed on 6 November 2025).

**Table 4:** Differential diagnosis of Lynch Syndrome: Hereditary cancer predisposition syndromes with increased risk for colorectal cancer.

*Gene(s)/* Inheritance	Disorder	Clinical Characteristics	Other Non-Gastrointestinal Characteristics
***APC*/AD**	*APC*-related disorders: Classic FAP (>100 polyps) Attenuated FAP (10–100 polyps) GAPPS	Numerous adenomatous polyps in the colon, often with a younger age of onset compared to Lynch syndrome CRC risk: ~100% if untreated Increased risk of medulloblastoma, thyroid papillary carcinoma, hepatoblastoma, pancreatic, gastric, and duodenal cancers	CHRPE Desmoids Epidermoid cysts Odontomas Osteomas Supernumerary teeth In attenuated FAP, extracolonic manifestations are less common. Desmoid tumors are associated with 3′ *APC* variants
***MUTYH*/AR**	*MUTYH*-associated polyposis: 10–100 colorectal polyps	Colonic adenomas, hyperplastic and/or serrated polyps, and duodenal adenomas CRC risk: 80–90% if left untreated. CRC may occur with no polyposis, and most CRCs are MSS	Benign adrenal lesions CHRPE Jawbone cysts Thyroid nodules
***PTEN*/AD**	*PTEN*-hamartoma tumor syndrome: Cowden syndrome Bannayan-Riley-Ruvalcaba syndrome Proteus-like syndrome	Numerous colorectal polyps: hamartomas, adenomas, ganglioneuromas, serrated polyps, juvenile polyps, inflammatory polyps, and lymphoid aggregates CRC risk: 2- to 3-fold increased lifetime risk	Increased risk of breast, thyroid, and renal cancers. Clinical criteria for the diagnosis of this condition have been developed.
***BMPR1A*** ***SMAD4*** **AD**	Juvenile polyposis syndrome (JPS)	Multiple juvenile polyps (hamartomatous) in the gastrointestinal tract (5 or more) CRC risk: ~68% by age 60.	Individuals with PV in *SMAD4* are at risk of Hereditary Hemorrhagic Telangiectasia (HHT)
***AXIN2*/AD** ***GREM1*/AD *MBD4*/AD+AR *MLH3*/AR** ***MSH3*/AR *NTHL1*/AR *POLD1*/AD *POLE*/AD**	Oligopolyposis syndromes (10–100 polyps): *AXIN2*-oligodontia-colorectal cancer syndrome *GREM1*-Hereditary mixed polyposis syndrome *MBD4* biallelic pathogenic variants/*MBD4*-associated neoplasia syndrome *MLH3*-associated polyposis syndrome *MSH3*-associated polyposis syndrome *NTHL1*-biallelic pathogenic variants *POLD1/POLE*-Polymerase Proofreading associated Polyposis (PPAP)	*AXIN2:* tubular adenomas (0–100) CRC risk: increased, not well-defined. *GREM1:* Multiple polyps of more than 1 histologic type. CRC risk: 11–20% *MBD4:* 15–100 tubular adenomas. CRC risk: increased, not well-defined. *MLH3* and *MSH3*: 30–100 tubular adenomas. CRC risk: increased, not well-defined. *NTHL1:* 1–100 polyps Adenomas most frequently. CRC risk: >20% *POLD1/POLE:* 30–100 tubular adenomas. CRC risk: >20%	*AXIN2:* hypo- or oligodontia *MBD4:* biallelic (acute myeloid leukemia); biallelic and monoallelic (uveal melanoma) *NTHL1:* Breast cancer is most common, endometrial malignancies, urothelial carcinomas, brain tumors, hematologic malignancies, basal cell carcinomas, head and neck squamous cell carcinomas, and cervical cancers in multiple individuals. *POLD1/POLE:* other cancers reported with limited evidence.
***RNF43*/AD**	Serrated polyposis syndrome (SPS)	Polyps: 5–100 serrated polyps/lesions (any histological subtype) CRC risk: increased, not well-defined	PVs in *RNF43* have been identified as a rare cause of SPS.
***RPS20*/AD**	*RPS20*-associated hereditary nonpolyposis CRC	MMR proficient tumors CRC risk: increased, not well-defined	
***STK11*/AD**	Peutz-Jeghers syndrome (PJS)	≥2 Peutz-Jeghers-type hamartomatous polyps (colon and small intestine) CRC risk: ~39% lifetime risk	Increased risk for breast, pancreatic, stomach, small intestine, lung, testicular, and gynecologic cancers
***TP53*/AD**	Li-Fraumeni syndrome (LFS)	CRC risk: >20%	Increased risk for sarcoma, breast, brain, leukemia, lung, adrenocortical, and other cancers

Abbreviations: FAP, familial adenomatous polyposis; GAPPS, gastric adenocarcinoma and proximal polyposis of the stomach; CHRPE, congenital hypertrophy of the retinal pigment epithelium; CRC, colorectal cancer; MMR, mismatch repair; AD, autosomal dominant; AR, autosomal recessive. Created in BioRender. Eroglu S. (2025). https://BioRender.com/0jov3ns (accessed on 6 November 2025).

## Supplementary Materials

The following supporting information can be downloaded at https://www.mdpi.com/article/10.3390/cancers17243981/s1. Figure S1: Model of the human mismatch repair (MMR) process.

## References

[1] Warthin A.S. (1913). Heredity with reference to carcinoma. Arch. Intern. Med..

[2] Lynch H.T., Shaw M.W., Magnuson C.W., Larsen A.L., Krush A.J. (1966). Hereditary factors in cancer. Study of two large midwestern kindreds. Arch. Intern. Med..

[3] Lynch H.T., Krush A.J. (1971). The cancer family syndrome and cancer control. Surg. Gynecol. Obstet..

[4] Boland C.R., Troncale F.J. (1984). Familial colonic cancer without antecedent polyposis. Ann. Intern. Med..

[5] Win A.K., Jenkins M.A., Dowty J.G., Antoniou A.C., Lee A., Giles G.G., Buchanan D.D., Clendenning M., Rosty C., Ahnen D.J. (2017). Prevalence and Penetrance of Major Genes and Polygenes for Colorectal Cancer. Cancer Epidemiol. Biomarkers Prev..

[6] Lynch H.T., Lynch P.M., Lanspa S.J., Snyder C.L., Lynch J.F., Boland C.R. (2009). Review of the Lynch syndrome: History, molecular genetics, screening, differential diagnosis, and medicolegal ramifications. Clin. Genet..

[7] Kahn R.M., Gordhandas S., Maddy B.P., Baltich Nelson B., Askin G., Christos P.J., Caputo T.A., Chapman-Davis E., Holcomb K., Frey M.K. (2019). Universal endometrial cancer tumor typing: How much has immunohistochemistry, microsatellite instability, and MLH1 methylation improved the diagnosis of Lynch syndrome across the population?. Cancer.

[8] Hitchins M.P., Ward R.L. (2009). Constitutional (germline) MLH1 epimutation as an aetiological mechanism for hereditary non-polyposis colorectal cancer. J. Med. Genet..

[9] Ortega J., Lee G.S., Gu L., Yang W., Li G.M. (2021). Mispair-bound human MutS-MutL complex triggers DNA incisions and activates mismatch repair. Cell Res..

[10] Li G.M. (2008). Mechanisms and functions of DNA mismatch repair. Cell Res..

[11] Kunkel T.A., Erie D.A. (2005). DNA mismatch repair. Annu. Rev. Biochem..

[12] Nolano A., Medugno A., Trombetti S., Liccardo R., De Rosa M., Izzo P., Duraturo F. (2022). Hereditary Colorectal Cancer: State of the Art in Lynch Syndrome. Cancers.

[13] Olave M.C., Graham R.P. (2022). Mismatch repair deficiency: The what, how and why it is important. Genes. Chromosomes Cancer.

[14] Cerretelli G., Ager A., Arends M.J., Frayling I.M. (2020). Molecular pathology of Lynch syndrome. J. Pathol..

[15] Kim T.M., Laird P.W., Park P.J. (2013). The landscape of microsatellite instability in colorectal and endometrial cancer genomes. Cell.

[16] Ijsselsteijn R., van Hees S., Drost M., Jansen J.G., de Wind N. (2022). Induction of mismatch repair deficiency, compromised DNA damage signaling and compound hypermutagenesis by a dietary mutagen in a cell-based model for Lynch syndrome. Carcinogenesis.

[17] Witt D., Faust U., Strobl-Wildemann G., Sturm M., Buchert R., Zuleger T., Admard J., Casadei N., Ossowski S., Haack T.B. (2023). Genome sequencing identifies complex structural MLH1 variant in unsolved Lynch syndrome. Mol. Genet. Genomic Med..

[18] Pinto D., Pinto C., Guerra J., Pinheiro M., Santos R., Vedeld H.M., Yohannes Z., Peixoto A., Santos C., Pinto P. (2018). Contribution of MLH1 constitutional methylation for Lynch syndrome diagnosis in patients with tumor MLH1 downregulation. Cancer Med..

[19] Sipila L.J., Aavikko M., Ravantti J., Martin S., Kuopio T., Lahtinen L., Gen F., Peltomaki P., Mecklin J.P., Aaltonen L.A. (2024). Detection of a major Lynch Syndrome-causing MLH1 founder variant in a large-scale genotyped cohort. Fam. Cancer.

[20] Cajal A.R., Pinero T.A., Verzura A., Santino J.P., Solano A.R., Kalfayan P.G., Ferro A., Vaccaro C. (2016). Founder mutation in Lynch syndrome. Medicina.

[21] Dominguez-Valentin M., Sampson J.R., Seppala T.T., Ten Broeke S.W., Plazzer J.P., Nakken S., Engel C., Aretz S., Jenkins M.A., Sunde L. (2020). Cancer risks by gene, age, and gender in 6350 carriers of pathogenic mismatch repair variants: Findings from the Prospective Lynch Syndrome Database. Genet. Med..

[22] Dominguez-Valentin M., Plazzer J.P., Sampson J.R., Engel C., Aretz S., Jenkins M.A., Sunde L., Bernstein I., Capella G., Balaguer F. (2021). No Difference in Penetrance between Truncating and Missense/Aberrant Splicing Pathogenic Variants in MLH1 and MSH2: A Prospective Lynch Syndrome Database Study. J. Clin. Med..

[23] Clendenning M., Hampel H., LaJeunesse J., Lindblom A., Lockman J., Nilbert M., Senter L., Sotamaa K., de la Chapelle A. (2006). Long-range PCR facilitates the identification of PMS2-specific mutations. Hum. Mutat..

[24] Tomsic J., Senter L., Liyanarachchi S., Clendenning M., Vaughn C.P., Jenkins M.A., Hopper J.L., Young J., Samowitz W., de la Chapelle A. (2013). Recurrent and founder mutations in the PMS2 gene. Clin. Genet..

[25] Raskin L., Schwenter F., Freytsis M., Tischkowitz M., Wong N., Chong G., Narod S.A., Levine D.A., Bogomolniy F., Aronson M. (2011). Characterization of two Ashkenazi Jewish founder mutations in MSH6 gene causing Lynch syndrome. Clin. Genet..

[26] Rhees J., Arnold M., Boland C.R. (2014). Inversion of exons 1-7 of the MSH2 gene is a frequent cause of unexplained Lynch syndrome in one local population. Fam. Cancer.

[27] Ligtenberg M.J., Kuiper R.P., Geurts van Kessel A., Hoogerbrugge N. (2013). EPCAM deletion carriers constitute a unique subgroup of Lynch syndrome patients. Fam. Cancer.

[28] Thompson B.A., Spurdle A.B., Plazzer J.P., Greenblatt M.S., Akagi K., Al-Mulla F., Bapat B., Bernstein I., Capella G., den Dunnen J.T. (2014). Application of a 5-tiered scheme for standardized classification of 2,360 unique mismatch repair gene variants in the InSiGHT locus-specific database. Nat. Genet..

[29] Landrum M.J., Lee J.M., Riley G.R., Jang W., Rubinstein W.S., Church D.M., Maglott D.R. (2014). ClinVar: Public archive of relationships among sequence variation and human phenotype. Nucleic Acids Res..

[30] Rath A., Radecki A.A., Rahman K., Gilmore R.B., Hudson J.R., Cenci M., Tavtigian S.V., Grady J.P., Heinen C.D. (2022). A calibrated cell-based functional assay to aid classification of MLH1 DNA mismatch repair gene variants. Hum. Mutat..

[31] Jia X., Burugula B.B., Chen V., Lemons R.M., Jayakody S., Maksutova M., Kitzman J.O. (2021). Massively parallel functional testing of MSH2 missense variants conferring Lynch syndrome risk. Am. J. Hum. Genet..

[32] Frederiksen J.H., Jensen S.B., Tumer Z., Hansen T.V.O. (2021). Classification of MSH6 Variants of Uncertain Significance Using Functional Assays. Int. J. Mol. Sci..

[33] Rayner E., Tiersma Y., Fortuno C., van Hees-Stuivenberg S., Drost M., Thompson B., Spurdle A.B., de Wind N. (2022). Predictive functional assay-based classification of PMS2 variants in Lynch syndrome. Hum. Mutat..

[34] Vasen H.F., Mecklin J.P., Khan P.M., Lynch H.T. (1991). The International Collaborative Group on Hereditary Non-Polyposis Colorectal Cancer (ICG-HNPCC). Dis. Colon. Rectum.

[35] Vasen H.F., Watson P., Mecklin J.P., Lynch H.T. (1999). New clinical criteria for hereditary nonpolyposis colorectal cancer (HNPCC, Lynch syndrome) proposed by the International Collaborative group on HNPCC. Gastroenterology.

[36] Vasen H.F., Moslein G., Alonso A., Bernstein I., Bertario L., Blanco I., Burn J., Capella G., Engel C., Frayling I. (2007). Guidelines for the clinical management of Lynch syndrome (hereditary non-polyposis cancer). J. Med. Genet..

[37] Idos G., Valle L., Adam M.P., Feldman J., Mirzaa G.M., Pagon R.A., Wallace S.E., Amemiya A. (1993). Lynch Syndrome. GeneReviews((R)).

[38] Lindor N.M., Rabe K., Petersen G.M., Haile R., Casey G., Baron J., Gallinger S., Bapat B., Aronson M., Hopper J. (2005). Lower cancer incidence in Amsterdam-I criteria families without mismatch repair deficiency: Familial colorectal cancer type X. J. Am. Med. Assoc..

[39] Nieminen T.T., Abdel-Rahman W.M., Ristimaki A., Lappalainen M., Lahermo P., Mecklin J.P., Jarvinen H.J., Peltomaki P. (2011). BMPR1A mutations in hereditary nonpolyposis colorectal cancer without mismatch repair deficiency. Gastroenterology.

[40] Nieminen T.T., O’Donohue M.F., Wu Y., Lohi H., Scherer S.W., Paterson A.D., Ellonen P., Abdel-Rahman W.M., Valo S., Mecklin J.P. (2014). Germline mutation of RPS20, encoding a ribosomal protein, causes predisposition to hereditary nonpolyposis colorectal carcinoma without DNA mismatch repair deficiency. Gastroenterology.

[41] Schulz E., Klampfl P., Holzapfel S., Janecke A.R., Ulz P., Renner W., Kashofer K., Nojima S., Leitner A., Zebisch A. (2014). Germline variants in the SEMA4A gene predispose to familial colorectal cancer type X. Nat. Commun..

[42] Martin-Morales L., Feldman M., Vershinin Z., Garre P., Caldes T., Levy D. (2017). SETD6 dominant negative mutation in familial colorectal cancer type X. Hum. Mol. Genet..

[43] Garre P., Martin L., Sanz J., Romero A., Tosar A., Bando I., Llovet P., Diaque P., Garcia-Paredes B., Diaz-Rubio E. (2015). BRCA2 gene: A candidate for clinical testing in familial colorectal cancer type X. Clin. Genet..

[44] Kim I.J., Ku J.L., Kang H.C., Park J.H., Yoon K.A., Shin Y., Park H.W., Jang S.G., Lim S.K., Han S.Y. (2004). Mutational analysis of OGG1, MYH, MTH1 in FAP, HNPCC and sporadic colorectal cancer patients: R154H OGG1 polymorphism is associated with sporadic colorectal cancer patients. Hum. Genet..

[45] Segui N., Mina L.B., Lazaro C., Sanz-Pamplona R., Pons T., Navarro M., Bellido F., Lopez-Doriga A., Valdes-Mas R., Pineda M. (2015). Germline Mutations in FAN1 Cause Hereditary Colorectal Cancer by Impairing DNA Repair. Gastroenterology.

[46] Nejadtaghi M., Jafari H., Farrokhi E., Samani K.G. (2017). Familial Colorectal Cancer Type X (FCCTX) and the correlation with various genes-A systematic review. Curr. Probl. Cancer.

[47] Garcia F.A.O., de Andrade E.S., de Campos Reis Galvao H., da Silva Sabato C., Campacci N., de Paula A.E., Evangelista A.F., Santana I.V.V., Melendez M.E., Reis R.M. (2022). New insights on familial colorectal cancer type X syndrome. Sci. Rep..

[48] Xu Y., Li C., Zhang Y., Guo T., Zhu C., Xu Y., Liu F. (2020). Comparison Between Familial Colorectal Cancer Type X and Lynch Syndrome: Molecular, Clinical, and Pathological Characteristics and Pedigrees. Front. Oncol..

[49] Haraldsdottir S., Hampel H., Tomsic J., Frankel W.L., Pearlman R., de la Chapelle A., Pritchard C.C. (2014). Colon and endometrial cancers with mismatch repair deficiency can arise from somatic, rather than germline, mutations. Gastroenterology.

[50] Pearlman R., Frankel W.L., Swanson B.J., Jones D., Zhao W., Yilmaz A., Miller K., Bacher J., Bigley C., Nelsen L. (2021). Prospective Statewide Study of Universal Screening for Hereditary Colorectal Cancer: The Ohio Colorectal Cancer Prevention Initiative. JCO Precis. Oncol..

[51] Pastrello C., Fornasarig M., Pin E., Berto E., Pivetta B., Viel A. (2009). Somatic mosaicism in a patient with Lynch syndrome. Am. J. Med. Genet. A.

[52] Sourrouille I., Coulet F., Lefevre J.H., Colas C., Eyries M., Svrcek M., Bardier-Dupas A., Parc Y., Soubrier F. (2013). Somatic mosaicism and double somatic hits can lead to MSI colorectal tumors. Fam. Cancer.

[53] Geurts-Giele W.R., Rosenberg E.H., Rens A.V., Leerdam M.E.V., Dinjens W.N., Bleeker F.E. (2019). Somatic mosaicism by a de novo MLH1 mutation as a cause of Lynch syndrome. Mol. Genet. Genom. Med..

[54] Guillerm E., Svrcek M., Bardier-Dupas A., Basset N., Coulet F., Colas C. (2020). Molecular tumor testing in patients with Lynch-like syndrome reveals a de novo mosaic variant of a mismatch repair gene transmitted to offspring. Eur. J. Hum. Genet..

[55] O’Brien A., Macfarlane S., Sommerlad M., Schirwani S. (2024). Mosaic Muir Torre Syndrome: Keratoacanthoma as a Piece of the Puzzle. Am. J. Dermatopathol..

[56] Walker R., Clendenning M., Joo J.E., Xue J., Mahmood K., Georgeson P., Como J., Joseland S., Preston S.G., Chan J.M. (2023). A mosaic pathogenic variant in MSH6 causes MSH6-deficient colorectal and endometrial cancer in a patient classified as suspected Lynch syndrome: A case report. Fam. Cancer.

[57] Holter S., Hall M.J., Hampel H., Jasperson K., Kupfer S.S., Larsen Haidle J., Mork M.E., Palaniapppan S., Senter L., Stoffel E.M. (2022). Risk assessment and genetic counseling for Lynch syndrome—Practice resource of the National Society of Genetic Counselors and the Collaborative Group of the Americas on Inherited Gastrointestinal Cancer. J. Genet. Couns..

[58] Tung N., Ricker C., Messersmith H., Balmana J., Domchek S., Stoffel E.M., Almhanna K., Arun B., Chavarri-Guerra Y., Cohen S.A. (2024). Selection of Germline Genetic Testing Panels in Patients With Cancer: ASCO Guideline. J. Clin. Oncol..

[59] (2025). Referenced with Permission from the NCCN Clinical Practice Guidelines in Oncology (NCCN Guidelines^®^) for Guideline Genetic/Familial High-Risk Assessment: Colorectal, Endometrial, and Gastric V.1.2025. © National Comprehensive Cancer Network, Inc. https://www.nccn.org/.

[60] Hamilton S.R., Liu B., Parsons R.E., Papadopoulos N., Jen J., Powell S.M., Krush A.J., Berk T., Cohen Z., Tetu B. (1995). The molecular basis of Turcot’s syndrome. N. Engl. J. Med..

[61] Kruse R., Rutten A., Lamberti C., Hosseiny-Malayeri H.R., Wang Y., Ruelfs C., Jungck M., Mathiak M., Ruzicka T., Hartschuh W. (1998). Muir-Torre phenotype has a frequency of DNA mismatch-repair-gene mutations similar to that in hereditary nonpolyposis colorectal cancer families defined by the Amsterdam criteria. Am. J. Hum. Genet..

[62] Weissman S.M., Bellcross C., Bittner C.C., Freivogel M.E., Haidle J.L., Kaurah P., Leininger A., Palaniappan S., Steenblock K., Vu T.M. (2011). Genetic counseling considerations in the evaluation of families for Lynch syndrome—A review. J. Genet. Couns..

[63] Rajan N., Cook S., Best K., Lynch Syndrome U.K.S.C.g., Monahan K. (2024). Universal testing of cutaneous sebaceous carcinoma: A missed opportunity in Lynch syndrome detection. Lancet Oncol..

[64] PMS2 Gene [updated 2025 May 2025]. https://medlineplus.gov/genetics/gene/pms2/.

[65] Colas C., Guerrini-Rousseau L., Suerink M., Gallon R., Kratz C.P., Ayuso E., Group E.G.C.G., Brugieres L., Wimmer K. (2024). ERN GENTURIS guidelines on constitutional mismatch repair deficiency diagnosis, genetic counselling, surveillance, quality of life, and clinical management. Eur. J. Hum. Genet..

[66] Jacquart A., Depas M., McFarland R., Basel D. Unknown Synergistic Effect of Digenetic Inheritance of MMR Pathogenic Mutations: Double Heterozygosity in Lynch Syndrome, A Single Case Report & Family Study. Proceedings of the 2016 NSGC 35th Annual Education Conference.

[67] Rodriguez-Bigas M.A., Boland C.R., Hamilton S.R., Henson D.E., Jass J.R., Khan P.M., Lynch H., Perucho M., Smyrk T., Sobin L. (1997). A National Cancer Institute Workshop on Hereditary Nonpolyposis Colorectal Cancer Syndrome: Meeting highlights and Bethesda guidelines. J. Natl. Cancer Inst..

[68] Syngal S., Fox E.A., Eng C., Kolodner R.D., Garber J.E. (2000). Sensitivity and specificity of clinical criteria for hereditary non-polyposis colorectal cancer associated mutations in MSH2 and MLH1. J. Med. Genet..

[69] Umar A., Boland C.R., Terdiman J.P., Syngal S., de la Chapelle A., Ruschoff J., Fishel R., Lindor N.M., Burgart L.J., Hamelin R. (2004). Revised Bethesda Guidelines for hereditary nonpolyposis colorectal cancer (Lynch syndrome) and microsatellite instability. J. Natl. Cancer Inst..

[70] Hampel H., Frankel W.L., Martin E., Arnold M., Khanduja K., Kuebler P., Clendenning M., Sotamaa K., Prior T., Westman J.A. (2008). Feasibility of screening for Lynch syndrome among patients with colorectal cancer. J. Clin. Oncol..

[71] Bartley A.N., Mills A.M., Konnick E., Overman M., Ventura C.B., Souter L., Colasacco C., Stadler Z.K., Kerr S., Howitt B.E. (2022). Mismatch Repair and Microsatellite Instability Testing for Immune Checkpoint Inhibitor Therapy: Guideline From the College of American Pathologists in Collaboration With the Association for Molecular Pathology and Fight Colorectal Cancer. Arch. Pathol. Lab. Med..

[72] Moreira L., Balaguer F., Lindor N., de la Chapelle A., Hampel H., Aaltonen L.A., Hopper J.L., Le Marchand L., Gallinger S., Newcomb P.A. (2012). Identification of Lynch syndrome among patients with colorectal cancer. J. Am. Med. Assoc..

[73] Ranganathan M., Sacca R.E., Trottier M., Maio A., Kemel Y., Salo-Mullen E., Catchings A., Kane S., Wang C., Ravichandran V. (2023). Prevalence and Clinical Implications of Mismatch Repair-Proficient Colorectal Cancer in Patients With Lynch Syndrome. JCO Precis. Oncol..

[74] Evaluation of Genomic Applications in Practice and Prevention (EGAPP) Working Group (2009). Recommendations from the EGAPP Working Group: Genetic testing strategies in newly diagnosed individuals with colorectal cancer aimed at reducing morbidity and mortality from Lynch syndrome in relatives. Genet. Med..

[75] Palomaki G.E., McClain M.R., Melillo S., Hampel H.L., Thibodeau S.N. (2009). EGAPP supplementary evidence review: DNA testing strategies aimed at reducing morbidity and mortality from Lynch syndrome. Genet. Med..

[76] Balmana J., Balaguer F., Cervantes A., Arnold D., Group E.G.W. (2013). Familial risk-colorectal cancer: ESMO Clinical Practice Guidelines. Ann. Oncol..

[77] Giardiello F.M., Allen J.I., Axilbund J.E., Boland C.R., Burke C.A., Burt R.W., Church J.M., Dominitz J.A., Johnson D.A., Kaltenbach T. (2014). Guidelines on genetic evaluation and management of Lynch syndrome: A consensus statement by the US Multi-Society Task Force on colorectal cancer. Gastroenterology.

[78] Provenzale D., Gupta S., Ahnen D.J., Bray T., Cannon J.A., Cooper G., David D.S., Early D.S., Erwin D., Ford J.M. (2016). Genetic/Familial High-Risk Assessment: Colorectal Version 1.2016, NCCN Clinical Practice Guidelines in Oncology. J. Natl. Compr. Cancer Netw..

[79] Goodfellow P.J., Billingsley C.C., Lankes H.A., Ali S., Cohn D.E., Broaddus R.J., Ramirez N., Pritchard C.C., Hampel H., Chassen A.S. (2015). Combined Microsatellite Instability, MLH1 Methylation Analysis, and Immunohistochemistry for Lynch Syndrome Screening in Endometrial Cancers from GOG210: An NRG Oncology and Gynecologic Oncology Group Study. J. Clin. Oncol..

[80] FDA Grants Accelerated Approval to Pembrolizumab for First Tissue/Site Agnostic Indication. https://www.fda.gov/drugs/informationondrugs/approveddrugs/ucm560040.htm.

[81] Latham A., Srinivasan P., Kemel Y., Shia J., Bandlamudi C., Mandelker D., Middha S., Hechtman J., Zehir A., Dubard-Gault M. (2019). Microsatellite Instability Is Associated With the Presence of Lynch Syndrome Pan-Cancer. J. Clin. Oncol..

[82] Papadopoulou E., Rigas G., Fountzilas E., Boutis A., Giassas S., Mitsimponas N., Daliani D., Ziogas D.C., Liontos M., Ramfidis V. (2024). Microsatellite Instability Is Insufficiently Used as a Biomarker for Lynch Syndrome Testing in Clinical Practice. JCO Precis. Oncol..

[83] Kastrinos F., Uno H., Ukaegbu C., Alvero C., McFarland A., Yurgelun M.B., Kulke M.H., Schrag D., Meyerhardt J.A., Fuchs C.S. (2017). Development and Validation of the PREMM(5) Model for Comprehensive Risk Assessment of Lynch Syndrome. J. Clin. Oncol..

[84] Chen S., Wang W., Lee S., Nafa K., Lee J., Romans K., Watson P., Gruber S.B., Euhus D., Kinzler K.W. (2006). Prediction of germline mutations and cancer risk in the Lynch syndrome. J. Am. Med. Assoc..

[85] Barnetson R.A., Tenesa A., Farrington S.M., Nicholl I.D., Cetnarskyj R., Porteous M.E., Campbell H., Dunlop M.G. (2006). Identification and survival of carriers of mutations in DNA mismatch-repair genes in colon cancer. N. Engl. J. Med..

[86] Sandoval R.L., Horiguchi M., Ukaegbu C., Furniss C.S., Uno H., Syngal S., Yurgelun M.B. (2023). PREMM5 distinguishes sporadic from Lynch syndrome-associated MMR-deficient/MSI-high colorectal cancer. Fam. Cancer.

[87] Heald B., Hampel H., Church J., Dudley B., Hall M.J., Mork M.E., Singh A., Stoffel E., Stoll J., You Y.N. (2020). Collaborative Group of the Americas on Inherited Gastrointestinal Cancer Position statement on multigene panel testing for patients with colorectal cancer and/or polyposis. Fam. Cancer.

[88] Hitchins M.P., Damaso E., Alvarez R., Zhou L., Hu Y., Diniz M.A., Pineda M., Capella G., Pearlman R., Hampel H. (2023). Constitutional MLH1 Methylation Is a Major Contributor to Mismatch Repair-Deficient, MLH1-Methylated Colorectal Cancer in Patients Aged 55 Years and Younger. J. Natl. Compr. Cancer Netw..

[89] Lee K., Abul-Husn N.S., Amendola L.M., Brothers K.B., Chung W.K., Gollob M.H., Gordon A.S., Harrison S.M., Hershberger R.E., Li M. (2025). ACMG SF v3.3 list for reporting of secondary findings in clinical exome and genome sequencing: A policy statement of the American College of Medical Genetics and Genomics (ACMG). Genet. Med..

[90] Melo M., Ribeiro M., Silva P.F., Valente S., Alves F., Venancio M., Sequeiros J., Freixo J.P., Antunes D., Oliveira J. (2025). Medically Actionable Secondary Findings from Whole-Exome Sequencing (WES) Data in a Sample of 3972 Individuals. Int. J. Mol. Sci..

[91] Seppala T.T., Latchford A., Negoi I., Sampaio Soares A., Jimenez-Rodriguez R., Sanchez-Guillen L., Evans D.G., Ryan N., Crosbie E.J., Dominguez-Valentin M. (2021). European guidelines from the EHTG and ESCP for Lynch syndrome: An updated third edition of the Mallorca guidelines based on gene and gender. Br. J. Surg..

[92] Tomita N., Ishida H., Tanakaya K., Yamaguchi T., Kumamoto K., Tanaka T., Hinoi T., Miyakura Y., Hasegawa H., Takayama T. (2021). Japanese Society for Cancer of the Colon and Rectum (JSCCR) guidelines 2020 for the Clinical Practice of Hereditary Colorectal Cancer. Int. J. Clin. Oncol..

[93] Stjepanovic N., Moreira L., Carneiro F., Balaguer F., Cervantes A., Balmana J., Martinelli E., on behalf of the ESMO Guidelines Committee (2019). Hereditary gastrointestinal cancers: ESMO Clinical Practice Guidelines for diagnosis, treatment and follow-updagger. Ann. Oncol..

[94] Herzig D.O., Buie W.D., Weiser M.R., You Y.N., Rafferty J.F., Feingold D., Steele S.R. (2017). Clinical Practice Guidelines for the Surgical Treatment of Patients With Lynch Syndrome. Dis. Colon. Rectum.

[95] Vasen H.F. (2005). Clinical description of the Lynch syndrome [hereditary nonpolyposis colorectal cancer (HNPCC)]. Fam. Cancer.

[96] Young J., Simms L.A., Biden K.G., Wynter C., Whitehall V., Karamatic R., George J., Goldblatt J., Walpole I., Robin S.A. (2001). Features of colorectal cancers with high-level microsatellite instability occurring in familial and sporadic settings: Parallel pathways of tumorigenesis. Am. J. Pathol..

[97] Jass J.R., Walsh M.D., Barker M., Simms L.A., Young J., Leggett B.A. (2002). Distinction between familial and sporadic forms of colorectal cancer showing DNA microsatellite instability. Eur. J. Cancer.

[98] Buttin B.M., Powell M.A., Mutch D.G., Rader J.S., Herzog T.J., Gibb R.K., Huettner P., Edmonston T.B., Goodfellow P.J. (2004). Increased risk for hereditary nonpolyposis colorectal cancer-associated synchronous and metachronous malignancies in patients with microsatellite instability-positive endometrial carcinoma lacking MLH1 promoter methylation. Clin. Cancer Res..

[99] Rijcken F.E., Hollema H., Kleibeuker J.H. (2002). Proximal adenomas in hereditary non-polyposis colorectal cancer are prone to rapid malignant transformation. Gut.

[100] Mecklin J.P., Sipponen P., Jarvinen H.J. (1986). Histopathology of colorectal carcinomas and adenomas in cancer family syndrome. Dis. Colon. Rectum.

[101] Lynch H.T., Smyrk T.C., Watson P., Lanspa S.J., Lynch J.F., Lynch P.M., Cavalieri R.J., Boland C.R. (1993). Genetics, natural history, tumor spectrum, and pathology of hereditary nonpolyposis colorectal cancer: An updated review. Gastroenterology.

[102] Ahadova A., von Knebel Doeberitz M., Blaker H., Kloor M. (2016). CTNNB1-mutant colorectal carcinomas with immediate invasive growth: A model of interval cancers in Lynch syndrome. Fam. Cancer.

[103] Vasen H.F.A. (2022). Progress Report: New insights into the prevention of CRC by colonoscopic surveillance in Lynch syndrome. Fam. Cancer.

[104] Miyaki M., Iijima T., Kimura J., Yasuno M., Mori T., Hayashi Y., Koike M., Shitara N., Iwama T., Kuroki T. (1999). Frequent mutation of beta-catenin and APC genes in primary colorectal tumors from patients with hereditary nonpolyposis colorectal cancer. Cancer Res..

[105] Johnson V., Volikos E., Halford S.E., Eftekhar Sadat E.T., Popat S., Talbot I., Truninger K., Martin J., Jass J., Houlston R. (2005). Exon 3 beta-catenin mutations are specifically associated with colorectal carcinomas in hereditary non-polyposis colorectal cancer syndrome. Gut.

[106] Ahadova A., Gallon R., Gebert J., Ballhausen A., Endris V., Kirchner M., Stenzinger A., Burn J., von Knebel Doeberitz M., Blaker H. (2018). Three molecular pathways model colorectal carcinogenesis in Lynch syndrome. Int. J. Cancer.

[107] de Miranda N.F., Goudkade D., Jordanova E.S., Tops C.M., Hes F.J., Vasen H.F., van Wezel T., Morreau H. (2012). Infiltration of Lynch colorectal cancers by activated immune cells associates with early staging of the primary tumor and absence of lymph node metastases. Clin. Cancer Res..

[108] Bauer K., Nelius N., Reuschenbach M., Koch M., Weitz J., Steinert G., Kopitz J., Beckhove P., Tariverdian M., von Knebel Doeberitz M. (2013). T cell responses against microsatellite instability-induced frameshift peptides and influence of regulatory T cells in colorectal cancer. Cancer Immunol. Immunother..

[109] Akiyama Y., Iwanaga R., Saitoh K., Shiba K., Ushio K., Ikeda E., Iwama T., Nomizu T., Yuasa Y. (1997). Transforming growth factor beta type II receptor gene mutations in adenomas from hereditary nonpolyposis colorectal cancer. Gastroenterology.

[110] Sanchez A., Bujanda L., Cuatrecasas M., Bofill A., Alvarez-Urturi C., Hernandez G., Aguilera L., Carballal S., Llach J., Herrera-Pariente C. (2021). Identification of Lynch Syndrome Carriers among Patients with Small Bowel Adenocarcinoma. Cancers.

[111] Suerink M., Kilinc G., Terlouw D., Hristova H., Sensuk L., van Egmond D., Farina Sarasqueta A., Langers A.M.J., van Wezel T., Morreau H. (2021). Prevalence of mismatch repair deficiency and Lynch syndrome in a cohort of unselected small bowel adenocarcinomas. J. Clin. Pathol..

[112] Aparicio T., Henriques J., Manfredi S., Tougeron D., Bouche O., Pezet D., Piessen G., Coriat R., Zaanan A., Legoux J.L. (2020). Small bowel adenocarcinoma: Results from a nationwide prospective ARCAD-NADEGE cohort study of 347 patients. Int. J. Cancer.

[113] Latham A., Shia J., Patel Z., Reidy-Lagunes D.L., Segal N.H., Yaeger R., Ganesh K., Connell L., Kemeny N.E., Kelsen D.P. (2021). Characterization and Clinical Outcomes of DNA Mismatch Repair-deficient Small Bowel Adenocarcinoma. Clin. Cancer Res..

[114] Lin C.F., Carwana H.E., Jiang S.F., Li D. (2024). Risk of Gastric and Small Intestinal Cancer in Patients With Lynch Syndrome: Data From a Large, Community-Based US Population. Clin. Transl. Gastroenterol..

[115] Kim J., Braun D., Ukaegbu C., Dhingra T.G., Kastrinos F., Parmigiani G., Syngal S., Yurgelun M.B. (2020). Clinical Factors Associated with Gastric Cancer in Individuals with Lynch Syndrome. Clin. Gastroenterol. Hepatol..

[116] Kumar S., Dudzik C.M., Reed M., Long J.M., Wangensteen K.J., Katona B.W. (2020). Upper Endoscopic Surveillance in Lynch Syndrome Detects Gastric and Duodenal Adenocarcinomas. Cancer Prev. Res..

[117] Ladigan-Badura S., Vangala D.B., Engel C., Bucksch K., Hueneburg R., Perne C., Nattermann J., Steinke-Lange V., Rahner N., Schackert H.K. (2021). Value of upper gastrointestinal endoscopy for gastric cancer surveillance in patients with Lynch syndrome. Int. J. Cancer.

[118] Caspers I.A., Eikenboom E.L., Lopez-Yurda M., van Grieken N.C.T., Bisseling T.M., Dekker E., Bastiaansen B.A.J., Cats A., van Leerdam M.E., Netherlands Foundation for Detection of Hereditary Tumours Collaborative I. (2024). Gastric and duodenal cancer in individuals with Lynch syndrome: A nationwide cohort study. EClinicalMedicine.

[119] Farha N., Hrabe J., Sleiman J., Beard J., Lyu R., Bhatt A., Church J., Heald B., Liska D., Mankaney G. (2022). Clinically actionable findings on surveillance EGD in asymptomatic patients with Lynch syndrome. Gastrointest. Endosc..

[120] Lu K.H., Dinh M., Kohlmann W., Watson P., Green J., Syngal S., Bandipalliam P., Chen L.M., Allen B., Conrad P. (2005). Gynecologic cancer as a “sentinel cancer” for women with hereditary nonpolyposis colorectal cancer syndrome. Obstet. Gynecol..

[121] Meyer L.A., Broaddus R.R., Lu K.H. (2009). Endometrial cancer and Lynch syndrome: Clinical and pathologic considerations. Cancer Control.

[122] Post C.C.B., Stelloo E., Smit V., Ruano D., Tops C.M., Vermij L., Rutten T.A., Jurgenliemk-Schulz I.M., Lutgens L., Jobsen J.J. (2021). Prevalence and Prognosis of Lynch Syndrome and Sporadic Mismatch Repair Deficiency in Endometrial Cancer. J. Natl. Cancer Inst..

[123] Broaddus R.R., Lynch H.T., Chen L.M., Daniels M.S., Conrad P., Munsell M.F., White K.G., Luthra R., Lu K.H. (2006). Pathologic features of endometrial carcinoma associated with HNPCC: A comparison with sporadic endometrial carcinoma. Cancer.

[124] Ketabi Z., Bartuma K., Bernstein I., Malander S., Gronberg H., Bjorck E., Holck S., Nilbert M. (2011). Ovarian cancer linked to Lynch syndrome typically presents as early-onset, non-serous epithelial tumors. Gynecol. Oncol..

[125] ACOG (2014). Practice Bulletin No. 147: Lynch syndrome. Obstet. Gynecol..

[126] Underkofler K.A., Ring K.L. (2023). Updates in gynecologic care for individuals with lynch syndrome. Front. Oncol..

[127] Weiss N.S., Sayvetz T.A. (1980). Incidence of endometrial cancer in relation to the use of oral contraceptives. N. Engl. J. Med..

[128] Vereide A.B., Arnes M., Straume B., Maltau J.M., Orbo A. (2003). Nuclear morphometric changes and therapy monitoring in patients with endometrial hyperplasia: A study comparing effects of intrauterine levonorgestrel and systemic medroxyprogesterone. Gynecol. Oncol..

[129] Baker J., Obermair A., Gebski V., Janda M. (2012). Efficacy of oral or intrauterine device-delivered progestin in patients with complex endometrial hyperplasia with atypia or early endometrial adenocarcinoma: A meta-analysis and systematic review of the literature. Gynecol. Oncol..

[130] Dashti S.G., Chau R., Ouakrim D.A., Buchanan D.D., Clendenning M., Young J.P., Winship I.M., Arnold J., Ahnen D.J., Haile R.W. (2015). Female Hormonal Factors and the Risk of Endometrial Cancer in Lynch Syndrome. J. Am. Med. Assoc..

[131] Liu Q., Simin J., Debelius J., Fall K., Sadr-Azodi O., Engstrand L., Williams C., Brusselaers N. (2021). Menopausal hormone therapies and risk of colorectal cancer: A Swedish matched-cohort study. Aliment. Pharmacol. Ther..

[132] (2024). Referenced with Permission from the NCCN Clinical Practice Guidelines in Oncology (NCCN Guidelines^®^) for Guideline Uterine Neoplasms V.3.2024. © National Comprehensive Cancer Network, Inc. https://www.nccn.org/.

[133] (2024). Referenced with Permission from the NCCN Clinical Practice Guidelines in Oncology (NCCN Guidelines^®^) for Guideline Ovarian Cancer V.3.2024. © National Comprehensive Cancer Network, Inc. https://www.nccn.org/.

[134] Wischhusen J.W., Ukaegbu C., Dhingra T.G., Uno H., Kastrinos F., Syngal S., Yurgelun M.B. (2020). Clinical Factors Associated with Urinary Tract Cancer in Individuals with Lynch Syndrome. Cancer Epidemiol. Biomark. Prev..

[135] Metcalfe M.J., Petros F.G., Rao P., Mork M.E., Xiao L., Broaddus R.R., Matin S.F. (2018). Universal Point of Care Testing for Lynch Syndrome in Patients with Upper Tract Urothelial Carcinoma. J. Urol..

[136] Gayhart M.G., Johnson N., Paul A., Quillin J.M., Hampton L.J., Idowu M.O., Smith S.C. (2020). Universal Mismatch Repair Protein Screening in Upper Tract Urothelial Carcinoma. Am. J. Clin. Pathol..

[137] Coleman J.A., Clark P.E., Bixler B.R., Buckley D.I., Chang S.S., Chou R., Hoffman-Censits J., Kulkarni G.S., Matin S.F., Pierorazio P.M. (2023). Diagnosis and Management of Non-Metastatic Upper Tract Urothelial Carcinoma: AUA/SUO Guideline. J. Urol..

[138] Roupret M., Seisen T., Birtle A.J., Capoun O., Comperat E.M., Dominguez-Escrig J.L., Gurses Andersson I., Liedberg F., Mariappan P., Hugh Mostafid A. (2023). European Association of Urology Guidelines on Upper Urinary Tract Urothelial Carcinoma: 2023 Update. Eur. Urol..

[139] Cloyd J.M., Chun Y.S., Ikoma N., Vauthey J.N., Aloia T.A., Cuddy A., Rodriguez-Bigas M.A., Nancy You Y. (2018). Clinical and Genetic Implications of DNA Mismatch Repair Deficiency in Biliary Tract Cancers Associated with Lynch Syndrome. J. Gastrointest. Cancer.

[140] Takamizawa S., Morizane C., Tanabe N., Maruki Y., Kondo S., Hijioka S., Ueno H., Sugano K., Hiraoka N., Okusaka T. (2022). Clinical characteristics of pancreatic and biliary tract cancers associated with Lynch syndrome. J. Hepatobiliary Pancreat. Sci..

[141] Kanaya N., Aoki H., Morito T., Taniguchi F., Shigeyasu K., Tamura C., Sugano K., Akagi K., Ishida H., Tanakaya K. (2022). Clinical features of biliary tract cancer in Japanese individuals with Lynch syndrome. J. Gastrointest. Oncol..

[142] Moller P., Seppala T.T., Bernstein I., Holinski-Feder E., Sala P., Gareth Evans D., Lindblom A., Macrae F., Blanco I., Sijmons R.H. (2018). Cancer risk and survival in path_MMR carriers by gene and gender up to 75 years of age: A report from the Prospective Lynch Syndrome Database. Gut.

[143] O’Connor C.A., Harrold E., Lin D., Walch H., Gazzo A., Ranganathan M., Kane S., Keane F., Schoenfeld J., Moss D. (2024). Lynch Syndrome and Somatic Mismatch Repair Variants in Pancreas Cancer. JAMA Oncol..

[144] Goggins M., Overbeek K.A., Brand R., Syngal S., Del Chiaro M., Bartsch D.K., Bassi C., Carrato A., Farrell J., Fishman E.K. (2020). Management of patients with increased risk for familial pancreatic cancer: Updated recommendations from the International Cancer of the Pancreas Screening (CAPS) Consortium. Gut.

[145] Kunnackal John G., Das Villgran V., Caufield-Noll C., Giardiello F.M. (2022). Comparison of universal screening in major lynch-associated tumors: A systematic review of literature. Fam. Cancer.

[146] Gallon R., Gawthorpe P., Phelps R.L., Hayes C., Borthwick G.M., Santibanez-Koref M., Jackson M.S., Burn J. (2021). How Should We Test for Lynch Syndrome? A Review of Current Guidelines and Future Strategies. Cancers.

[147] Kattapuram M., Shabet C., Austin S., Jacobs M.F., Koeppe E., Smith E.H., Lowe L., Else T., Cha K.B. (2023). A retrospective cohort study of genetic referral and diagnosis of Lynch syndrome in patients with cutaneous sebaceous lesions. Fam. Cancer.

[148] Cook S., Pethick J., Kibbi N., Hollestein L., Lavelle K., de Vere Hunt I., Turnbull C., Rous B., Husain A., Burn J. (2023). Sebaceous carcinoma epidemiology, associated malignancies and Lynch/Muir-Torre syndrome screening in England from 2008 to 2018. J. Am. Acad. Dermatol..

[149] Kim H., Lim K.Y., Park J.W., Kang J., Won J.K., Lee K., Shim Y., Park C.K., Kim S.K., Choi S.H. (2022). Sporadic and Lynch syndrome-associated mismatch repair-deficient brain tumors. Lab. Investig..

[150] Benusiglio P.R., Elder F., Touat M., Perrier A., Sanson M., Colas C., Guerrini-Rousseau L., Tran D.T., Trabelsi N., Carpentier C. (2023). Mismatch Repair Deficiency and Lynch Syndrome Among Adult Patients With Glioma. JCO Precis. Oncol..

[151] Le D.T., Durham J.N., Smith K.N., Wang H., Bartlett B.R., Aulakh L.K., Lu S., Kemberling H., Wilt C., Luber B.S. (2017). Mismatch repair deficiency predicts response of solid tumors to PD-1 blockade. Science.

[152] Oka S., Urakami S., Hagiwara K., Hayashida M., Sakaguchi K., Miura Y., Inoshita N., Arai M. (2023). The prevalence of lynch syndrome (DNA mismatch repair protein deficiency) in patients with primary localized prostate cancer using immunohistochemistry screening. Hered. Cancer Clin. Pract..

[153] Bancroft E.K., Page E.C., Brook M.N., Thomas S., Taylor N., Pope J., McHugh J., Jones A.B., Karlsson Q., Merson S. (2021). A prospective prostate cancer screening programme for men with pathogenic variants in mismatch repair genes (IMPACT): Initial results from an international prospective study. Lancet Oncol..

[154] Roberts M.E., Jackson S.A., Susswein L.R., Zeinomar N., Ma X., Marshall M.L., Stettner A.R., Milewski B., Xu Z., Solomon B.D. (2018). MSH6 and PMS2 germ-line pathogenic variants implicated in Lynch syndrome are associated with breast cancer. Genet. Med..

[155] Harkness E.F., Barrow E., Newton K., Green K., Clancy T., Lalloo F., Hill J., Evans D.G. (2015). Lynch syndrome caused by MLH1 mutations is associated with an increased risk of breast cancer: A cohort study. J. Med. Genet..

[156] Engel C., Loeffler M., Steinke V., Rahner N., Holinski-Feder E., Dietmaier W., Schackert H.K., Goergens H., von Knebel Doeberitz M., Goecke T.O. (2012). Risks of less common cancers in proven mutation carriers with lynch syndrome. J. Clin. Oncol..

[157] Stoll J., Rosenthal E., Cummings S., Willmott J., Bernhisel R., Kupfer S.S. (2020). No Evidence of Increased Risk of Breast Cancer in Women With Lynch Syndrome Identified by Multigene Panel Testing. JCO Precis. Oncol..

[158] Kaczmar J.M., Everett J., Ruth K., Martinez Stoffel E., Stoll J., Kupfer S., Hampel H., Stadler Z.K., Gaddam P., Rybak C. Sarcoma: A Lynch syndrome (LS)-associated malignancy?. Proceedings of the 2015 ASCO Annual Meeting I.

[159] Fummey E., Navarro P., Plazzer J.P., Frayling I.M., Knott S., Tenesa A. (2024). Estimating cancer risk in carriers of Lynch syndrome variants in UK Biobank. J. Med. Genet..

[160] Poumeaud F., Valentin T., Vande Perre P., Jaffrelot M., Bonnet D., Chibon F., Chevreau C., Selves J., Guimbaud R., Fares N. (2023). Special features of sarcomas developed in patients with Lynch syndrome: A systematic review. Crit. Rev. Oncol. Hematol..

[161] de Angelis de Carvalho N., Niitsuma B.N., Kozak V.N., Costa F.D., de Macedo M.P., Kupper B.E.C., Silva M.L.G., Formiga M.N., Volc S.M., Aguiar Junior S. (2020). Clinical and Molecular Assessment of Patients with Lynch Syndrome and Sarcomas Underpinning the Association with MSH2 Germline Pathogenic Variants. Cancers.

[162] Poumeaud F., Valentin T., Fares N., Segier B., Watson S., Verret B., Tlemsani C., Penel N., Lejeune S., Firmin N. (2025). Sarcomas developed in patients with Lynch Syndrome are enriched in pleomorphic soft-tissue sarcomas and are sensitive to immunotherapy. Eur. J. Cancer.

[163] Shehata M.S., Lofftus S.Y., Park J.Y., Singh A.S., Federman N.C., Eilber F.C., Crompton J.G., McCaw T.R. (2024). Sarcoma in patients with Lynch syndrome and response to immunotherapy. J. Surg. Oncol..

[164] Kaur R.J., Pichurin P.N., Hines J.M., Singh R.J., Grebe S.K., Bancos I. (2019). Adrenal Cortical Carcinoma Associated With Lynch Syndrome: A Case Report and Review of Literature. J. Endocr. Soc..

[165] Ahuja K., Goudar R. (2024). A novel lynch syndrome kindred with hereditary adrenal cortical carcinoma. Cancer Genet..

[166] Raymond V.M., Everett J.N., Furtado L.V., Gustafson S.L., Jungbluth C.R., Gruber S.B., Hammer G.D., Stoffel E.M., Greenson J.K., Giordano T.J. (2013). Adrenocortical carcinoma is a lynch syndrome-associated cancer. J. Clin. Oncol..

[167] Domenech M., Grau E., Solanes A., Izquierdo A., Del Valle J., Carrato C., Pineda M., Duenas N., Pujol M., Lazaro C. (2021). Characteristics of Adrenocortical Carcinoma Associated With Lynch Syndrome. J. Clin. Endocrinol. Metab..

[168] Casey R.T., Giger O., Seetho I., Marker A., Pitfield D., Boyle L.H., Gurnell M., Shaw A., Tischkowitz M., Maher E.R. (2018). Rapid disease progression in a patient with mismatch repair-deficient and cortisol secreting adrenocortical carcinoma treated with pembrolizumab. Semin. Oncol..

[169] Bhattacharya P., Leslie S.W., McHugh T.W. (2025). Lynch Syndrome (Hereditary Nonpolyposis Colorectal Cancer). StatPearls.

[170] Schwitalle Y., Kloor M., Eiermann S., Linnebacher M., Kienle P., Knaebel H.P., Tariverdian M., Benner A., von Knebel Doeberitz M. (2008). Immune response against frameshift-induced neopeptides in HNPCC patients and healthy HNPCC mutation carriers. Gastroenterology.

[171] Reuschenbach M., Kloor M., Morak M., Wentzensen N., Germann A., Garbe Y., Tariverdian M., Findeisen P., Neumaier M., Holinski-Feder E. (2010). Serum antibodies against frameshift peptides in microsatellite unstable colorectal cancer patients with Lynch syndrome. Fam. Cancer.

[172] Ishikawa T., Fujita T., Suzuki Y., Okabe S., Yuasa Y., Iwai T., Kawakami Y. (2003). Tumor-specific immunological recognition of frameshift-mutated peptides in colon cancer with microsatellite instability. Cancer Res..

[173] Kloor M., Reuschenbach M., Pauligk C., Karbach J., Rafiyan M.R., Al-Batran S.E., Tariverdian M., Jager E., von Knebel Doeberitz M. (2020). A Frameshift Peptide Neoantigen-Based Vaccine for Mismatch Repair-Deficient Cancers: A Phase I/IIa Clinical Trial. Clin. Cancer Res..

[174] von Knebel Doeberitz M., Kloor M. (2013). Towards a vaccine to prevent cancer in Lynch syndrome patients. Fam. Cancer.

[175] Pardoll D.M. (2012). The blockade of immune checkpoints in cancer immunotherapy. Nat. Rev. Cancer.

[176] FDA Approves First Cancer Treatment for Any Solid Tumor with a Specific Genetic Feature. https://www.drugs.com/newdrugs/fda-approves-keytruda-pembrolizumab-first-cancer-any-solid-tumor-specific-genetic-feature-4538.html.

[177] FDA Approves Merck’s KEYTRUDA. (Pembrolizumab) for Adult and Pediatric Patients with Unresectable or Metastatic, Microsatellite Instability-High (MSI-H) or Mismatch Repair Deficient (dMMR) Solid Tumors. https://www.merck.com/news/fda-approves-mercks-keytruda-pembrolizumab-for-adult-and-pediatric-patients-with-unresectable-or-metastatic-microsatellite-instability-high-msi-h-or-mismatch-repair-deficient-cance/.

[178] Andre T., Shiu K.K., Kim T.W., Jensen B.V., Jensen L.H., Punt C., Smith D., Garcia-Carbonero R., Benavides M., Gibbs P. (2020). Pembrolizumab in Microsatellite-Instability-High Advanced Colorectal Cancer. N. Engl. J. Med..

[179] Overman M.J., McDermott R., Leach J.L., Lonardi S., Lenz H.J., Morse M.A., Desai J., Hill A., Axelson M., Moss R.A. (2017). Nivolumab in patients with metastatic DNA mismatch repair-deficient or microsatellite instability-high colorectal cancer (CheckMate 142): An open-label, multicentre, phase 2 study. Lancet Oncol..

[180] Andre T., Elez E., Van Cutsem E., Jensen L.H., Bennouna J., Mendez G., Schenker M., de la Fouchardiere C., Limon M.L., Yoshino T. (2024). Nivolumab plus Ipilimumab in Microsatellite-Instability-High Metastatic Colorectal Cancer. N. Engl. J. Med..

[181] Westin S.N., Moore K., Chon H.S., Lee J.Y., Thomes Pepin J., Sundborg M., Shai A., de la Garza J., Nishio S., Gold M.A. (2024). Durvalumab Plus Carboplatin/Paclitaxel Followed by Maintenance Durvalumab With or Without Olaparib as First-Line Treatment for Advanced Endometrial Cancer: The Phase III DUO-E Trial. J. Clin. Oncol..

[182] Patel S.A., Nilsson M.B., Le X., Cascone T., Jain R.K., Heymach J.V. (2023). Molecular Mechanisms and Future Implications of VEGF/VEGFR in Cancer Therapy. Clin. Cancer Res..

[183] Alabi F., Okpalanwaka I.F., Azoroh B., Okoyeocha E., Balogun T., Ayinde I.B. (2025). Lenvatinib Plus Pembrolizumab Versus Chemotherapy in Advanced Endometrial Cancer: Efficacy and Safety Insights. Cureus.

[184] Eskander R.N., Sill M.W., Beffa L., Moore R.G., Hope J.M., Musa F.B., Mannel R., Shahin M.S., Cantuaria G.H., Girda E. (2023). Pembrolizumab plus Chemotherapy in Advanced Endometrial Cancer. N. Engl. J. Med..

[185] Powell M.A., Bjorge L., Willmott L., Novak Z., Black D., Gilbert L., Sharma S., Valabrega G., Landrum L.M., Gropp-Meier M. (2024). Overall survival in patients with endometrial cancer treated with dostarlimab plus carboplatin-paclitaxel in the randomized ENGOT-EN6/GOG-3031/RUBY trial. Ann. Oncol..

[186] Makker V., Colombo N., Casado Herraez A., Santin A.D., Colomba E., Miller D.S., Fujiwara K., Pignata S., Baron-Hay S., Ray-Coquard I. (2022). Lenvatinib plus Pembrolizumab for Advanced Endometrial Cancer. N. Engl. J. Med..

[187] O’Malley D.M., Bariani G.M., Cassier P.A., Marabelle A., Hansen A.R., De Jesus Acosta A., Miller W.H., Safra T., Italiano A., Mileshkin L. (2022). Pembrolizumab in Patients With Microsatellite Instability-High Advanced Endometrial Cancer: Results From the KEYNOTE-158 Study. J. Clin. Oncol..

[188] Andre T., Berton D., Curigliano G., Sabatier R., Tinker A.V., Oaknin A., Ellard S., de Braud F., Arkenau H.T., Trigo J. (2023). Antitumor Activity and Safety of Dostarlimab Monotherapy in Patients With Mismatch Repair Deficient Solid Tumors: A Nonrandomized Controlled Trial. JAMA Netw. Open.

[189] Woerner S.M., Kloor M., Mueller A., Rueschoff J., Friedrichs N., Buettner R., Buzello M., Kienle P., Knaebel H.P., Kunstmann E. (2005). Microsatellite instability of selective target genes in HNPCC-associated colon adenomas. Oncogene.

[190] Willis J.A., Reyes-Uribe L., Chang K., Lipkin S.M., Vilar E. (2020). Immune Activation in Mismatch Repair-Deficient Carcinogenesis: More Than Just Mutational Rate. Clin. Cancer Res..

[191] Chang K., Taggart M.W., Reyes-Uribe L., Borras E., Riquelme E., Barnett R.M., Leoni G., San Lucas F.A., Catanese M.T., Mori F. (2018). Immune Profiling of Premalignant Lesions in Patients With Lynch Syndrome. JAMA Oncol..

[192] Koornstra J.J., de Jong S., Boersma-van Eck W., Zwart N., Hollema H., de Vries E.G., Kleibeuker J.H. (2009). Fas ligand expression in lynch syndrome-associated colorectal tumours. Pathol. Oncol. Res..

[193] Abidi A., Westdorp H., Gorris M.A.J., Scheijen B., Boller A.-L., Irusquieta C.B., Hoogerbrugge N., Schreibelt G., de Vries J.I.J.M. (2022). Dendritic cells to prevent cancer: Immune responses against neoantigens after dendritic cell vaccination of Lynch Syndrome patients. J. Immunol..

[194] Vilar E., Willis J., D’Alise M., Hall M., Cruz-Correa M., Idos G.E., Thirumurthi S., Leoni G., Garzia I., Antonucci L. (2024). 638 Nous-209 vaccine induces shared neoantigen immunogenicity for cancer interception in healthy lynch syndrome carriers: Results from phase Ib/II trial. J. Immunother. Cancer.

[195] Leoni G., D’Alise A.M., Cotugno G., Langone F., Garzia I., De Lucia M., Fichera I., Vitale R., Bignone V., Tucci F.G. (2020). A Genetic Vaccine Encoding Shared Cancer Neoantigens to Treat Tumors with Microsatellite Instability. Cancer Res..

[196] Berinstein N.L. (2002). Carcinoembryonic antigen as a target for therapeutic anticancer vaccines: A review. J. Clin. Oncol..

[197] Engel B.J., Bowser J.L., Broaddus R.R., Carson D.D. (2016). MUC1 stimulates EGFR expression and function in endometrial cancer. Oncotarget.

[198] Ramanathan R.K., Lee K.M., McKolanis J., Hitbold E., Schraut W., Moser A.J., Warnick E., Whiteside T., Osborne J., Kim H. (2005). Phase I study of a MUC1 vaccine composed of different doses of MUC1 peptide with SB-AS2 adjuvant in resected and locally advanced pancreatic cancer. Cancer Immunol. Immunother..

[199] Lepisto A.J., Moser A.J., Zeh H., Lee K., Bartlett D., McKolanis J.R., Geller B.A., Schmotzer A., Potter D.P., Whiteside T. (2008). A phase I/II study of a MUC1 peptide pulsed autologous dendritic cell vaccine as adjuvant therapy in patients with resected pancreatic and biliary tumors. Cancer Ther..

[200] Morse M.A., Chaudhry A., Gabitzsch E.S., Hobeika A.C., Osada T., Clay T.M., Amalfitano A., Burnett B.K., Devi G.R., Hsu D.S. (2013). Novel adenoviral vector induces T-cell responses despite anti-adenoviral neutralizing antibodies in colorectal cancer patients. Cancer Immunol. Immunother..

[201] Balint J.P., Gabitzsch E.S., Rice A., Latchman Y., Xu Y., Messerschmidt G.L., Chaudhry A., Morse M.A., Jones F.R. (2015). Extended evaluation of a phase 1/2 trial on dosing, safety, immunogenicity, and overall survival after immunizations with an advanced-generation Ad5 [E1-, E2b-]-CEA(6D) vaccine in late-stage colorectal cancer. Cancer Immunol. Immunother..

[202] Kimura T., McKolanis J.R., Dzubinski L.A., Islam K., Potter D.M., Salazar A.M., Schoen R.E., Finn O.J. (2013). MUC1 vaccine for individuals with advanced adenoma of the colon: A cancer immunoprevention feasibility study. Cancer Prev. Res..

[203] Gatti-Mays M.E., Redman J.M., Donahue R.N., Palena C., Madan R.A., Karzai F., Bilusic M., Sater H.A., Marte J.L., Cordes L.M. (2020). A Phase I Trial Using a Multitargeted Recombinant Adenovirus 5 (CEA/MUC1/Brachyury)-Based Immunotherapy Vaccine Regimen in Patients with Advanced Cancer. Oncologist.

[204] Schoen R.E., Boardman L.A., Cruz-Correa M., Bansal A., Kastenberg D., Hur C., Dzubinski L., Kaufman S.F., Rodriguez L.M., Richmond E. (2023). Randomized, Double-Blind, Placebo-Controlled Trial of MUC1 Peptide Vaccine for Prevention of Recurrent Colorectal Adenoma. Clin. Cancer Res..

[205] Bilusic M., McMahon S., Madan R.A., Karzai F., Tsai Y.T., Donahue R.N., Palena C., Jochems C., Marte J.L., Floudas C. (2021). Phase I study of a multitargeted recombinant Ad5 PSA/MUC-1/brachyury-based immunotherapy vaccine in patients with metastatic castration-resistant prostate cancer (mCRPC). J. Immunother. Cancer.

[206] Margolin K., Morishima C., Velcheti V., Miller J.S., Lee S.M., Silk A.W., Holtan S.G., Lacroix A.M., Fling S.P., Kaiser J.C. (2018). Phase I Trial of ALT-803, A Novel Recombinant IL15 Complex, in Patients with Advanced Solid Tumors. Clin. Cancer Res..

[207] Romee R., Cooley S., Berrien-Elliott M.M., Westervelt P., Verneris M.R., Wagner J.E., Weisdorf D.J., Blazar B.R., Ustun C., DeFor T.E. (2018). First-in-human phase 1 clinical study of the IL-15 superagonist complex ALT-803 to treat relapse after transplantation. Blood.

[208] Prince A.E., Roche M.I. (2014). Genetic information, non-discrimination, and privacy protections in genetic counseling practice. J. Genet. Couns..

[209] Willard L., Uhlmann W., Prince A.E.R., Blasco D., Pal S., Roberts J.S., Consortium I.W. (2025). The Genetic Information Nondiscrimination Act and workplace genetic testing: Knowledge and perceptions of employed adults in the United States. J. Genet. Couns..

[210] De Castro M., Biesecker L.G., Turner C., Brenner R., Witkop C., Mehlman M., Bradburne C., Green R.C. (2016). Genomic medicine in the military. NPJ Genom. Med..

[211] Baruch S., Hudson K. (2008). Civilian and military genetics: Nondiscrimination policy in a post-GINA world. Am. J. Hum. Genet..

[212] Kesserwan C., Friedman Ross L., Bradbury A.R., Nichols K.E. (2016). The Advantages and Challenges of Testing Children for Heritable Predisposition to Cancer. Am. Soc. Clin. Oncol. Educ. Book..

[213] Scollon S., Anglin A.K., Thomas M., Turner J.T., Wolfe Schneider K. (2017). A Comprehensive Review of Pediatric Tumors and Associated Cancer Predisposition Syndromes. J. Genet. Couns..

[214] Zelley K., Schienda J., Gallinger B., Kohlmann W.K., McGee R.B., Scollon S.R., Schneider K.W. (2024). Update on Genetic Counselor Practice and Recommendations for Pediatric Cancer Predisposition Evaluation and Surveillance. Clin. Cancer Res..

[215] Green R.C., Berg J.S., Grody W.W., Kalia S.S., Korf B.R., Martin C.L., McGuire A.L., Nussbaum R.L., O’Daniel J.M., Ormond K.E. (2013). ACMG recommendations for reporting of incidental findings in clinical exome and genome sequencing. Genet. Med..

[216] U.S. Department of Defense (2020). Medical Standards for Military Service: Retention. DoD Instruction 6130.03.

[217] U.S. Department of Defense (2018). Medical Standards for Military Service: Appointment, Enlistment, or Induction. DoD Instruction 6130.03.

