# Supplementary material for: Lynch Syndrome in Focus: A Multidisciplinary Review of Cancer Risk, Clinical Management, and Special Populations
Source: Cancers (Basel). 2025 Dec 13;17(24):3981. doi: 10.3390/cancers17243981 (PMC12730836; doi:10.3390/cancers17243981)
Supplement: Supplementary file 1 [file cancers-17-03981-s001.zip › Figure S1.pdf]

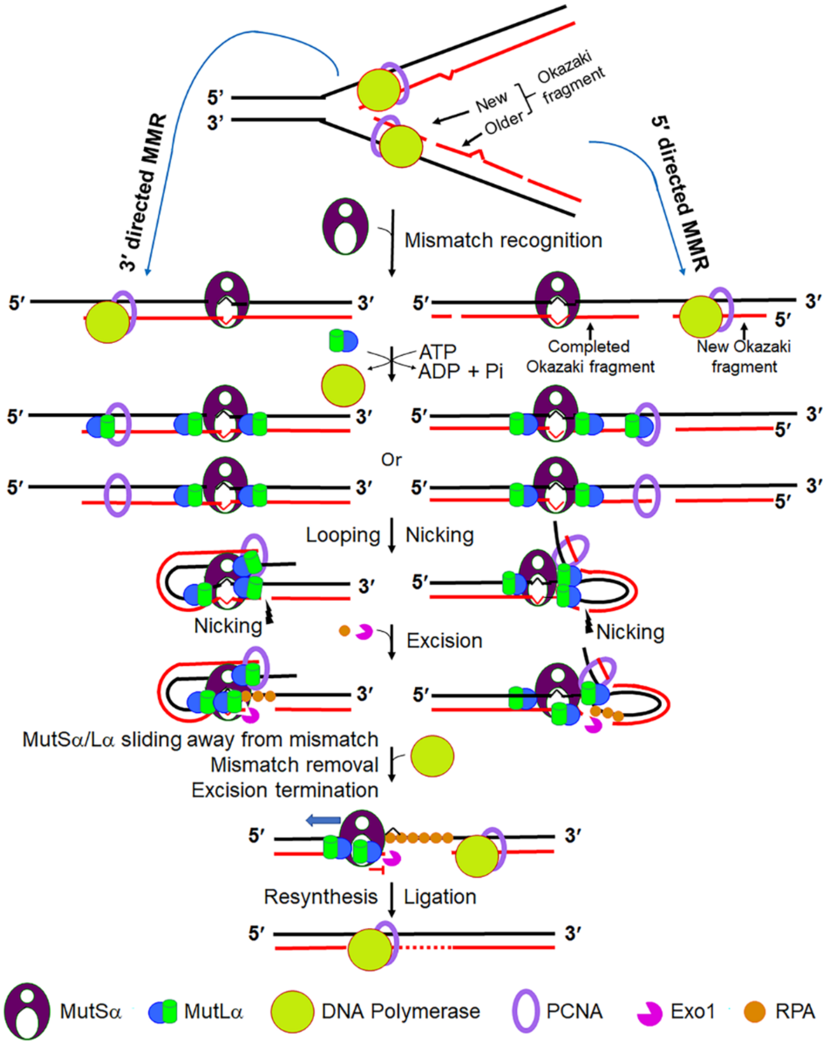

Figure S1. Model of the human mismatch repair (MMR) process.

Misincorporation can occur in either leading or lagging strand DNA synthesis, and these mispairs can be corrected in a 3'- or 5'-directed manner. Mismatch-bound MutSα recruits MutLα to DNA to form a stable initiation complex, in an ATP-dependent manner. In this ternary complex, mismatch-bound MutSα is flanked by MutLα molecules, and MutLα–MutLα or PCNA–MutLα/MutSα interactions bring mismatch and strand break to proximity, which simplifies the communication between the two sites. MutLα then makes a nick 5' to the mismatch on the nicked strand. Exo1 is recruited by MutLα to the nick and conducts 5' → 3' excision. Once the Exo1-catalyzed excision reaches the mismatch, MutSα or the MutSα–MutLα complex slides away from the mismatch, yielding the right of way to Exo1 for mismatch removal. The excision is terminated by the interactions between MutLα and Exo1. The DNA gap is filled by DNA polymerase δ in concerted reactions with PCNA and

RPA, and the nick is ligated by ligase I. This model applies to both 3' nick-directed (left panel) and 5' nick-directed (right panel) MMR.

Reproduced from [Ortega J, Lee GS, Gu L, Yang W, Li GM. Mispair-bound human MutS-MutL complex triggers DNA incisions and activates mismatch repair. Cell Res. 2021 May;31(5):542-553. doi: 10.1038/s41422-021-00468-y. Epub 2021 Jan 28. PMID: 33510387; PMCID: PMC8089094] under the terms of the Creative Commons Attribution 4.0 International License (http://creativecommons.org/licenses/by/4.0/). No changes were made.
