# Supplementary figures and images for: Lynch Syndrome in Focus: A Multidisciplinary Review of Cancer Risk, Clinical Management, and Special Populations
Source: Cancers (Basel). 2025 Dec 13;17(24):3981. doi: 10.3390/cancers17243981 (PMC12730836; doi:10.3390/cancers17243981)

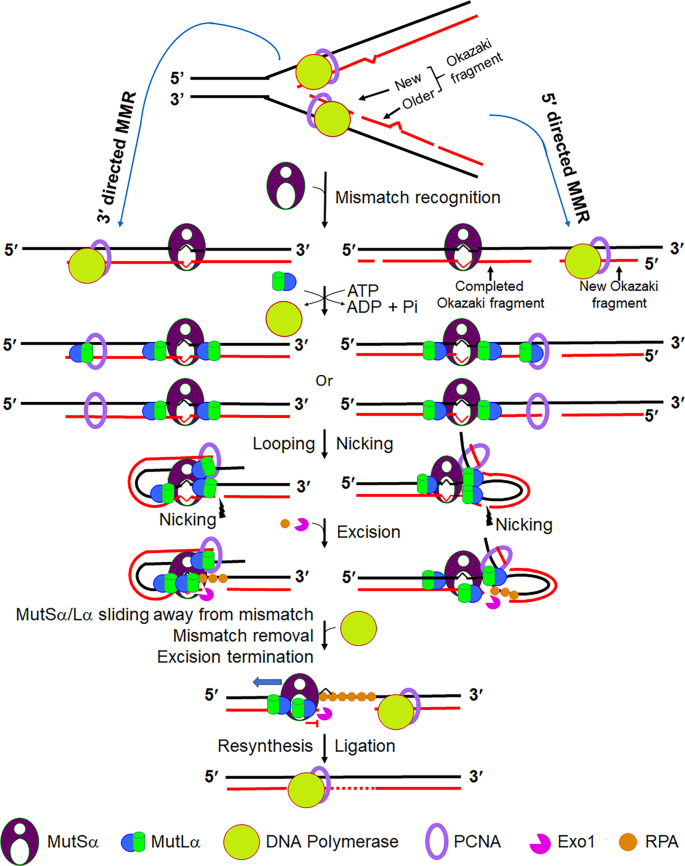

Supplement: Supplementary file 1 [file cancers-17-03981-s001.zip › supplementary figure 1.png]
